# Global Transcriptional Response to Heat Shock of the Legume Symbiont *Mesorhizobium loti* MAFF303099 Comprises Extensive Gene Downregulation

**DOI:** 10.1093/dnares/dst050

**Published:** 2013-11-25

**Authors:** Ana Alexandre, Marta Laranjo, Solange Oliveira

**Affiliations:** 1 ICAAM – Instituto de Ciências Agrárias e Ambientais Mediterrânicas (Laboratório de Microbiologia do Solo), Universidade de Évora, Núcleo da Mitra, Ap. 94, 7002-554 Évora, Portugal; 2 IIFA – Instituto de Investigação e Formação Avançada, Universidade de Évora, Ap. 94, 7002-554 Évora, Portugal

**Keywords:** stress, rhizobia, microarrays, chaperone, sHSP

## Abstract

Rhizobia, the bacterial legume symbionts able to fix atmospheric nitrogen inside root nodules, have to survive in varied environmental conditions. The aim of this study was to analyse the transcriptional response to heat shock of *Mesorhizobium loti* MAFF303099, a rhizobium with a large multipartite genome of 7.6 Mb that nodulates the model legume *Lotus japonicus*. Using microarray analysis, extensive transcriptomic changes were detected in response to heat shock: 30% of the protein-coding genes were differentially expressed (2067 genes in the chromosome, 62 in pMLa and 57 in pMLb). The highest-induced genes are in the same operon and code for two sHSP. Only one of the five *groEL* genes in MAFF303099 genome was induced by heat shock. Unlike other prokaryotes, the transcriptional response of this *Mesorhizobium* included the underexpression of an unusually large number of genes (72% of the differentially expressed genes). This extensive downregulation of gene expression may be an important part of the heat shock response, as a way of reducing energetic costs under stress. To our knowledge, this study reports the heat shock response of the largest prokaryote genome analysed so far, representing an important contribution to understand the response of plant-interacting bacteria to challenging environmental conditions.

## 1. Introduction

Rhizobia are soil bacteria able to colonize legume roots and form nodules, where atmospheric nitrogen is metabolized into compounds that can be used by the plant. The impact of the biological nitrogen fixation carried out by rhizobia in agriculture is both economic and environmental. Rhizobia may reduce the use of chemical N-fertilizers, which represent a production cost reduction and at the same time a decrease in the pollution resulting from N-fertilizers synthesis and from soil nitrate lixiviation.^1^

Rhizobia typically have large genomes, which are often composed by several replicons. These seem to be common features of bacterial species that interact with a host.^2^ This rhizobial trend to harbour a large accessory genome is probably related, not only to the symbiosis itself (interacting with a host), but also to the plasticity required to survive in complex and distinct environments. As free-living bacteria, rhizobia have to cope with changes in soil conditions and as plant-symbionts, rhizobia must overcome plant defence mechanisms and adapt to the intracellular nodule environment. For all the above reasons, these bacteria are particularly interesting to study stress response. The most important consequences of heat stress at the cellular level are protein denaturation and aggregation.^3^ These effects are common to other adverse conditions, as for example oxidative stress, so the study of the heat stress response is also relevant in understanding tolerance to other stresses.

The plasticity to respond to stressful conditions involves rapid changes in gene expression. Alternative sigma factors allow bacteria to rapidly redirect the RNA polymerases pool to the set of genes that are required to respond to a certain condition.^4^ Rhizobia genomes typically harbour a large number of alternative sigma factors, including multiple copies of *rpoH*, which encodes σ^32^, the major sigma factor involved in the heat shock response.^5^ σ^32^ might be involved in response to other stresses as seen in *Rhizobium etli*, where *rpoH2* seems to be more related to oxidative stress response.^6^ Furthermore, rhizobia with *rpoH* deletions may also be affected in their symbiotic phenotype.^6,7^ The transcription of ∼21% of the genes induced in response to a temperature upshift are *rpoH1* dependent in *Sinorhizobium meliloti* and these include chaperones, proteases and small heat shock proteins (sHSP).^5^

Important chaperone systems, such as GroES-GroEL and DnaK-DnaJ-GrpE, are σ^32^-regulated in most alphaproteobacteria. Chaperones play a key role in the heat shock response, as they are involved in promoting the acquisition of the native conformation by proteins that suffered denaturation and present the wrong folding.^8^ The importance of chaperonins in defining tolerance to temperature has been highlighted by several studies in *E. coli*.^9,10^ A more recent study showed that a high level of the GroESL system has a fundamental role in the evolution of heat tolerance.^11^ Some important reports on the functional analysis of the multiple *groESL* operons in rhizobia have been published.^12–14^ Mutational studies showed that *groESL* operons within the same genome are induced by different stimuli and that these genes are involved not only in stress tolerance, but also in the nodulation and nitrogen fixation processes.^15^ In *Mesorhizobium* spp., both *dnaK* and *groESL* genes were reported to be transcriptionally induced by a temperature upshift, especially in heat tolerant isolates.^16^ In rhizobia, *groESL* operons are often CIRCE (controlling inverted repeat of chaperone expression) regulated, as already reported in *Bradyrhizobium japonicum*, *S. meliloti* and *Rhizobium leguminosarum*.^13,17,18^ CIRCE is a highly conserved DNA sequence that serves as binding site of the repressor protein HrcA.^19,20^

Similar to the GroESL chaperonins, also the DnaKJ system seems to be involved in both heat tolerance and symbiosis phenotype.^21–23^ Regarding the co-chaperone *dnaJ*, rhizobia mutants showed that both stress tolerance and symbiotic performance are affected.^21,22,24^

sHSP are mostly involved in preventing the irreversible aggregation of misfolded proteins. The presence of a large number of sHSP is a common feature in rhizobia genomes.^25^ Some sHSP have a specific regulation designated by repression of heat shock gene expression (ROSE). ROSE element is a posttranscriptional regulation mechanism that consists in a conserved sequence downstream to the promoter.^26^

The heat shock response has been extensively studied in bacteria, however to our knowledge, only one rhizobia strain was studied in terms of heat shock transcriptome, namely *S. meliloti* 1021, a symbiont of *Medicago* spp.^5,27^ The strain analysed in the present report, *Mesorhizobium loti* MAFF303099, is a rhizobium able to establish nitrogen-fixing symbiosis with *Lotus* species.^28,29^ *M. loti* MAFF303099 genome comprises a large chromosome (7 Mb) and two plasmids designated as pMLa (352 kb) and pMLb (208 kb). A chromosomal symbiosis island (610 kb) contains most genes involved in nodulation and nitrogen fixation. A previous study showed that this strain is tolerant to heat shock and cold conditions, and grows well at pH 5.^30^

The aim of the present study is to characterize the transcriptional response to heat shock in a resourceful rhizobium with a large and complex genome. The analysis of the global transcriptional alterations following a sudden exposure to high-temperature conditions in *M. loti* MAFF303099 will contribute to a better understanding of the general stress response, in particular in symbiotic bacteria with multiple replicons and large accessory genome.

## 2. Materials and methods

### 2.1. RNA purification

Overnight cultures of *M. loti* MAFF303099 were grown in YMB^31^ at 28°C, to a final optical density of 0.3 (540 nm). A volume of 10 ml of bacterial culture was used in each treatment: 30 min at control (28°C) and heat shock (48°C) conditions. Cells were harvested and total RNA was purified using RNeasy Mini Kit (Qiagen). Contamination with DNA was removed by DNase digestion (Roche), followed by RNA cleanup using RNeasy Mini kit (Qiagen). Total RNA integrity was checked using the RNA Nano kit and an Agilent 2100 Bioanalyser (Agilent Technologies), while RNA quantification was performed using NanoDrop ND-1000 (NanoDrop Technologies). RNA was prepared from three independent cell cultures.

### 2.2. Microarray experiments

RNA processing as well as microarrays hybridization and raw data extraction were a service provided by Biocant Park—Genomics Unit (Portugal). In order to enrich the RNA samples in mRNA, the MICROB*Express*™ Kit (Ambion) was used to remove most of the rRNA. mRNA was then amplified with the MessageAmp™ II-Bacteria Kit (Ambion), with the incorporation of 5-(3-aminoallyl)-UTP (Ambion) for indirect labelling, which was carried out by the coupling of fluorescent Cy3 to the amplified RNA (aRNA), following the instructions of the Amino Allyl MessageAmp™ II aRNA Amplification Kit (Ambion).

The 40K array for *M. loti* MAFF303099 (MYcroarray) includes probes for 7231 genes (∼99% of the total number of protein-coding genes) with five replicates for each probe. Slide hybridization was carried out as described by the microarray's supplier, using the Gene Expression Hybridization Kit (Agilent Technologies). Data were acquired using a DNA Microarray B Scanner (Agilent Technologies), with an intensity of 100% PTM in the green channel.

### 2.3. Data analysis

The microarrays data were analysed using BRB ArrayTools (version 4.2).^32^ The arrays were normalized using the array median and genes that were differentially expressed following heat shock were identified using MeV software.^33^ Genes were considered differentially expressed for *P* ≤ 0.01 in the *t*-test.

Despite the recent update on the annotation of the MAFF303099 genome released by NCBI (October 2012), all genes differentially expressed that were annotated as ‘hypothetical protein’ were further analysed using Blast2GO software.^34^ This analysis included Blast, Mapping and Annotation, and allowed further annotation of many genes. In order to assign the highest number possible of genes to a clusters of orthologous genes (COG) category, STRING 9.0 database (search tool for the retrieval of interacting genes)^35^ was used.

MicrobesOnline Operon Predictions (www.microbesonline.org/operons/) was used for operon prediction.^36^ The identification of putative promoter sequences was performed using BPROM-Prediction of bacterial promoters software (www.softberry.com). DNAPlotter^37^ was used to generate circular DNA maps showing transcriptomics data.

Spearman's coefficient was used to test for correlation between genome size and number of over- or underexpressed genes (IBM SPSS Statistics, version 21).

### 2.4. Microarray data validation

Validation of microarray data was performed by real-time quantitative RT–PCR (qRT–PCR). cDNA was obtained by reverse transcription using Maxima First Strand cDNA Synthesis kit (Thermo Scientific) according to the manufacturer's instructions. Primers (Supplementary Table S1) were designed using Primer Express 3.0 software (Applied Biosystems). Real-time qRT–PCR reactions were prepared using 0.1 ng/µl of template cDNA, SYBR Green PCR Master Mix and 0.3 mM of each primer. Amplifications were carried out in a 7500 Real-time PCR System (Applied Biosystems). *C*_t_ values for the target genes were normalized using the reference genes *hisC*, *rpoA* and *sigA*, which showed no variation in the corresponding transcript levels for the experimental conditions used (data not shown).

## 3. Results and discussion

### 3.1. Global transcriptional response

**Figure 1.**
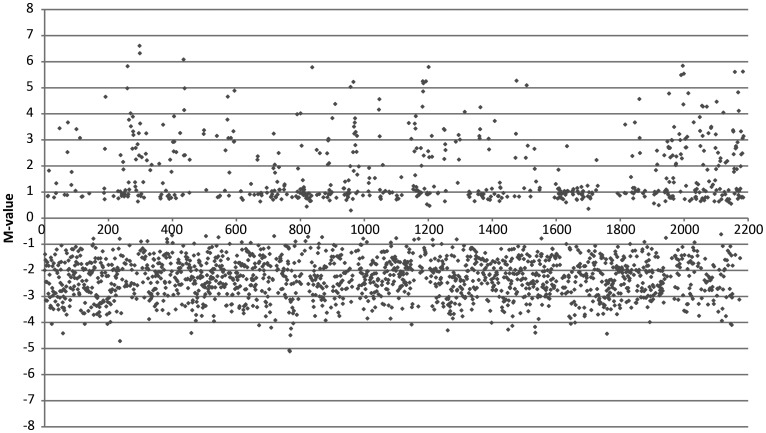
Microarrays analysis of *M. loti* MAFF303099 subjected to heat shock. *M*-values for the differentially expressed genes (*P* < 0.01) obtained from the comparison between heat shock (48°C) and control (28°C) conditions. Genes with increased amount of mRNA following the heat shock have positive *M*-values (overexpressed), while genes with decreased mRNA levels after heat shock show negative *M*-values (underexpressed).

Analysis of the *M. loti* MAFF303099 transcriptome allowed the identification of 2186 protein-coding genes that were differentially expressed after heat shock (out of 7231 genes analysed), with an average false discovery rate of 1.5% (accession number GSE43529). This indicates that the transcript levels of ∼30% of the protein-coding genes were altered by this stress. The transcriptional response included a much higher number of downregulated (1584) compared with the upregulated (602) genes (Fig. 1). The unexpected larger proportion of downregulated genes does not seem to be a feature of rhizobia, taking into account the similar numbers of induced and repressed genes reported for *S. meliloti*.^5,27^

**Figure 2.**
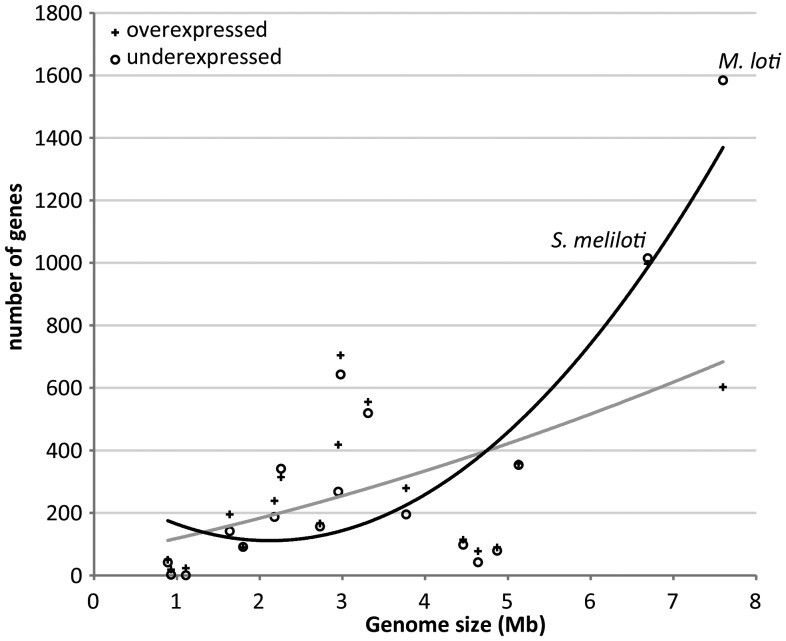
Number of overexpressed (+) and underexpressed (Ο) genes resulting from the transcriptome studies of the response to heat shock of 18 species of Bacteria and Archaea plotted against their genome size. Trendlines are shown in grey for the number of overexpressed genes (*R*^2^ = 0.35; Spearman's *ρ* = 0.583, *P* ≤ 0.05) and in black for the number of underexpressed genes (*R*^2^ = 0.69; Spearman's *ρ* = 0.608, *P* ≤ 0.01). From the smallest to the largest genome size: *Mycoplasma hyopneumoniae*^38^; *Tropheryma whip-plei*^39^; *Rickettsia prowazekii*^40^; *Campylobacter jejuni*^41^; *Strep-tococcus thermophilus*^42^; *Achaeoglobus fulgidus*^43^; *Bifidobacterium longum*^44^; *Xylella fastidiosa*^45^; *Listeria monocytogenes*^46^; *Acidithio-bacillus ferrooxidans*^47^; *Corynebacterium glutamicum*^48^; *Desul-fovibrio vulgaris*^49^; *Clostridium difficile*^50^; *Escherichia coli*^51^; *Methanosarcina barkeri*^52^; *Shewanella oneidensis*^53^; *S. meliloti*^5^; *M. loti* (this study). The two rhizobia species are denoted in the graphic. Note: in case of multiple heat shock transcriptome datasets for the same species, the dataset with the largest number of differentially expressed genes was chosen.

**Figure 3.**
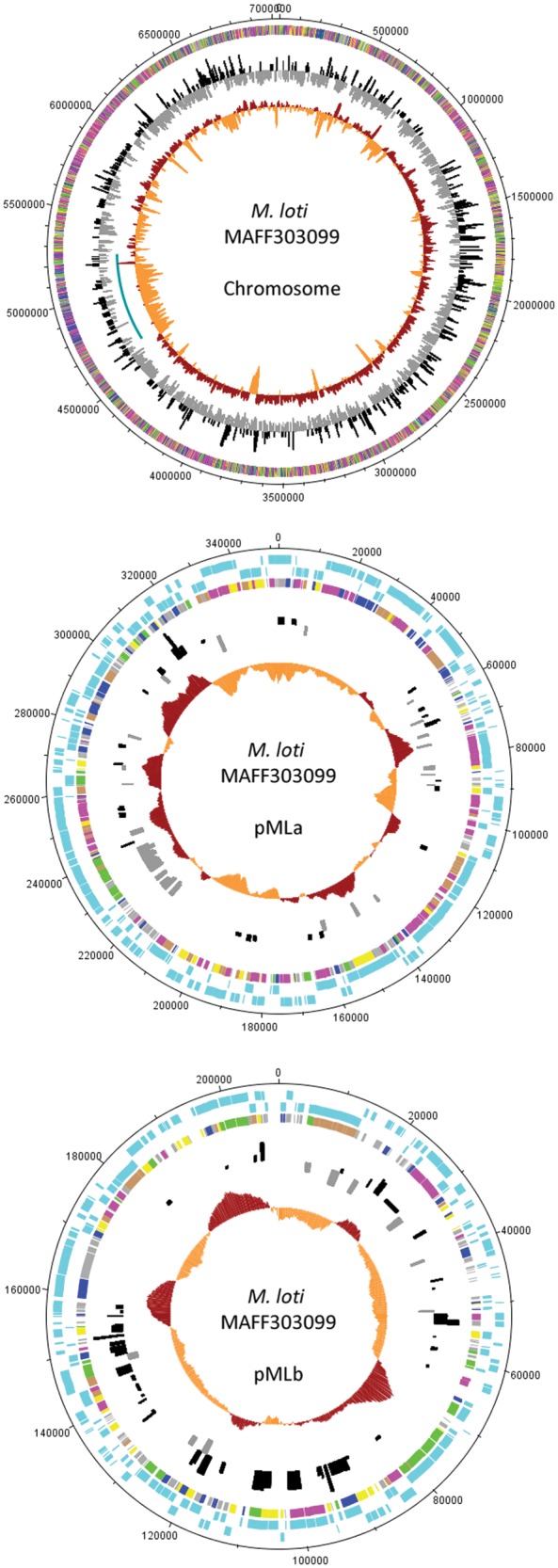
Circular plots of the chromosome and two plasmids included in *M. loti* MAFF303099 genome showing, from outer to inner rings: COG group for each gene; the heat shock transcriptome data (*M*-values) and the %GC plot. The plasmids plots include two additional outer rings displaying the genes encoded in the plus strand (outermost ring) and minus strand. COG colours: information storage and processing—blue; cellular processes and signalling—green; metabolism—magenta; poorly characterized—yellow; more than one COG category—brown; no COG—light grey. Transcriptome data: overexpressed—black; underexpressed—grey. %GC data: above average—dark red; below average—orange. The symbiosis island (coordinates 4 644 792–5 255 766)^28^ is marked in blue in the chromosome plot. This figure appears in colour in the online version of *DNA* Research.

**Figure 4.**
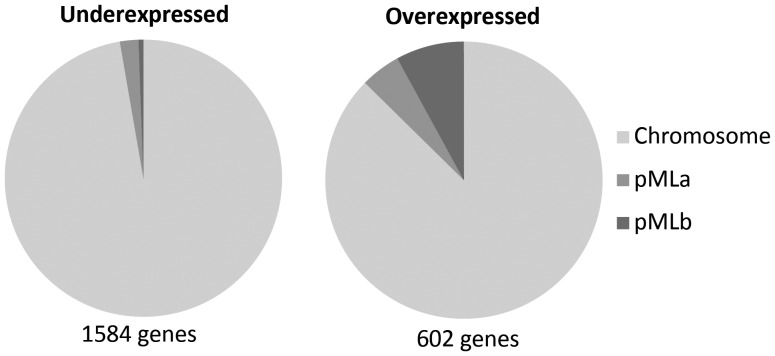
Number and location of differentially expressed genes in *M. loti* MAFF303099, following the heat shock.

To our knowledge, the present study reports the largest prokaryote genome studied so far in terms of response to heat shock. To investigate the influence of genome size in the global heat response, a comparison of the transcriptional response to heat of prokaryotes with different genome sizes was performed (Fig. 2). Strain MAFF303099 shows an unusual proportion of downregulated genes in response to heat shock compared with several other bacteria and archaea that, in general, show a similar number of genes under- and overexpressed following a temperature upshift (though different heat shock conditions are compared). Despite the fact that diverse species with distinct lifestyles and subjected to different heat shock conditions are compared in Fig. 2, analysis of the transcriptomic data suggests a general trend of pronounced increase in the number of downregulated genes with genome size. One might speculate that many expendable genes are shutdown, so that the cellular machinery can be more effective in the synthesis of the specific functional response. Nevertheless, the extensive gene downregulation is not particularly detected in the accessory genome that is presumably more dispensable. Indeed, the symbiosis island shows dispersed under- and overexpressed genes similar to the rest of the chromosome (Fig. 3). Furthermore, some highly induced genes are plasmid encoded, mainly in pMLb (Fig. 4). This is somewhat unexpected since symbiosis islands and plasmids are mobile elements in the genome, known to be laterally transferred within soil populations and thus less expected to carry genes essential for stress survival. In addition, the set of 100 genes with highest *M*-values comprises 14 plasmid encoded genes, while the 100 highly underexpressed genes are all chromosomal (Supplementary Table S2).

The high number of underexpressed genes may suggest that the heat shock response relies on a low-energy transcriptional response. Accordingly, ∼40% of the induced genes show a low increase in the transcriptional levels (*M* < 1). This low level of gene induction, commonly disregarded, may be important part of cells response, as pointed before by Wren and Conway.^54^

Analysis of the location of the differentially expressed genes in each replicon shows an apparently random distribution of over- and underexpressed genes, with the exception of an∼200 kb-long region located in 1 000 000–1 200 000 (462 genes) where all the differentially expressed genes are downregulated (Fig. 3). Both in the chromosome and plasmids, distribution of the differentially expressed genes seems to be unrelated to the DNA strand or GC content.

Real-time qRT–PCR was used to validate the microarray data. Genes were chosen based on *M*-values from the microarrays results, in order to include overexpressed, underexpressed and not differentially expressed genes, as well as genes encoded in both DNA strands and scattered in the chromosome. In general, the results from the real-time qRT–PCR experiments are in agreement with the microarrays analysis results (Table 1), with exception of the *dnaK* gene (discussed in the section ‘The DnaKJ chaperone system’).

**Table 1.** Microarrays data validation using real-time qRT–PCR

Locus tag	Gene	*M*-value	
		Real-time qRT–PCR	Microarrays
mll2386	–	14.6	6.6
mlr2394	*groEL*	11.9	5.8
mll1528	–	4.6	4.7
mll3429	*clpB*	7.0	2.9
mll3842	*citZ*	6.1	2.2
mlr5932	*acdS*	1.5	1.1
mll3873	–	−0.6	−1.9
mlr0883	*gcvT*	−0.9	−2.2
mlr6118	–	−2.4	−2.7
mll1546	*ftsZ*	−3.4	−3.7
mll6630	–	−3.8	−4.0
mlr2911	*flgB*	−3.7	−4.3
mll6578	*fixK*	−4.0	−5.1
mll6432	–	0.3	nde
mll4757	*dnaK*	5.5	nde
mll4755	*dnaJ*	−0.2	nde
mlr7618	*greA*	0.9	nde

nde, not differentially expressed.

**Figure 5.**
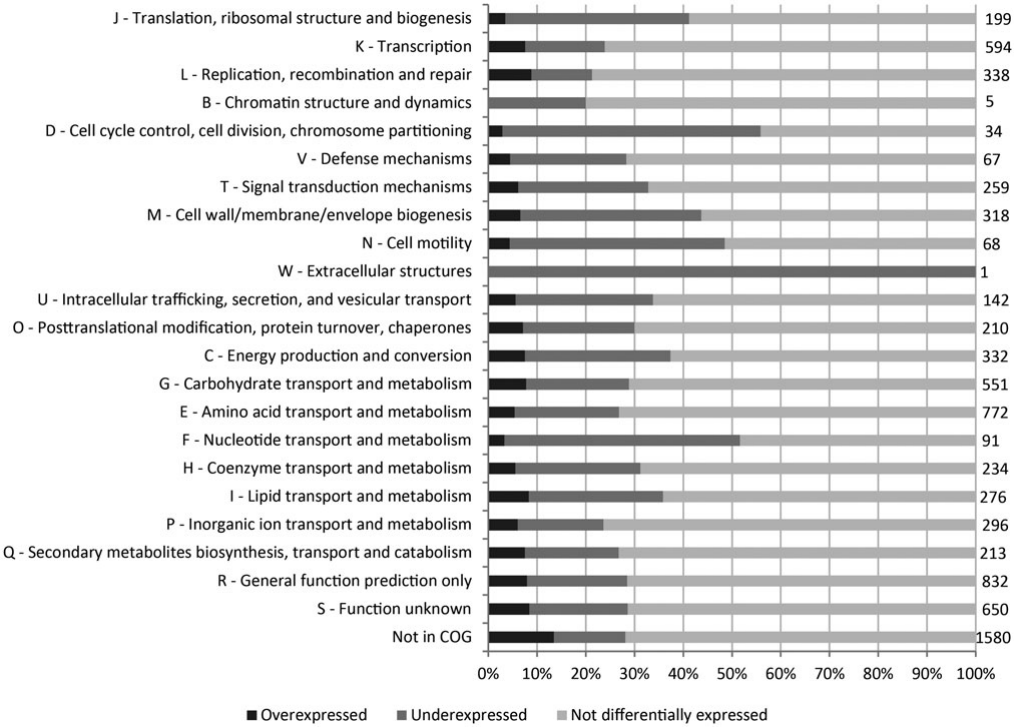
Percentage of genes from each COG category overexpressed and underexpressed after the heat shock. Genes not in a COG are also shown. The number of genes included in each category is shown at the right end of the graphic.

Protein-coding genes can be grouped into COG, according to their similarity in terms of domain architecture and function.^55^ The present study showed that temperature stress-induced changes in the expression of genes belonging to all COG categories from the MAFF303099 genome (Fig. 5). For all COG categories, the percentage of underexpressed genes is higher than that of overexpressed genes (Fig. 5 and Supplementary Fig. S1). In addition to the fact that a high number of differentially expressed genes are not in a COG (1580 genes), there are also many poorly characterized genes (‘S—function unknown’ and ‘R—general function prediction only’ categories) (Supplementary Fig. S1).

The COG category with the highest percentage of overexpressed genes is ‘L—replication, recombination and repair’ (9%). This COG category also shows the lowest percentage of underexpressed genes (12%). Nevertheless, the percentage of overexpressed genes is between 7 and 8% in nine other categories, including the COG category where chaperones and other heat shock proteins are included (‘O—posttranslational modification, protein turnover and chaperones’). This suggests a balanced response in terms of gene induction throughout the COG categories; yet, ∼13% of the overexpressed genes are not in a COG. Three categories include a high percentage of underexpressed genes following a heat shock, namely ‘D—cell cycle control, cell division, chromosome partitioning’, ‘F—nucleotide transport and metabolism’ and ‘N—cell motility’ (53, 48 and 44%, respectively). COG categories with a high number of overexpressed genes are ‘K—transcription’, ‘G—carbohydrate transport and metabolism’ and ‘E—amino acid transport and metabolism’ (Supplementary Fig. S1A). On the other hand, COG categories E and G also show a high number of underexpressed genes (Supplementary Fig. S1B). This is consistent with other bacterial species for which these two COG categories also showed a high number of over- and underexpressed genes in response to heat shock.^46,48^ According to Konstantinidis and Tiedje,^56^ large genomes tend to have a disproportional increase of genes belonging to COG ‘K—transcription’, ‘T—signal transduction mechanisms’ and ‘Q—secondary metabolites biosynthesis, transport and catabolism’, which could be expected to be the most underexpressed categories in large genome bacteria, nevertheless that is not observed in MAFF303099 (Fig. 5 and Supple-mentary Fig. S1B).

### 3.2. Small heat shock proteins

The two most heat shock-induced genes (mll2387 and mll2386 with *M*-values of 6.61 and 6.32, respectively) code for sHSP (Table 2). These genes are probably co-transcribed, since a single putative promoter was identified upstream mll2387 (predicted promoter: -35 TTGACG and -10 ACTCATTCT). This particular sHSP operon is likely to play an important role in the heat shock response, since homologous genes were also detected as the most overexpressed in *S. meliloti* following a less severe heat shock.^5^ Following a longer heat exposure, these genes seem to be less overexpressed, yet showing an induction of approximately 4-fold.^27^ The homologous *ibpAB* are also the most-induced genes in the heat shock response of *E. coli*.^51^ Western analysis of protein extracts of several rhizobia species confirmed an increase of the amount of sHPS with temperature upshifts.^25^ As in many other bacteria species, in *M. loti*, *S. meliloti* and *E. coli*, a ROSE element was identified upstream of these operons^26^ (Supplementary Fig. S2A). Nevertheless, these sHSP genes were reported as *rpoH*-dependent in *S. meliloti*,^5^ suggesting multiple regulation mechanisms that may allow a dynamic stress response.

**Table 2.** Overexpressed genes following the heat shock, identified by microarray analysis.

Locus tag	Replicon	COG category^a^	Gene description	*M*-value
mll2386	Chr	O	sHSP	6.61
mll2387	Chr	O	sHSP	6.32
mll3685	Chr	–	**PRC-barrel domain-containing protein**	6.08
msl2054	Chr	–	Hypothetical protein	5.84
mll1959	Chr	–	**BA14k family protein**	5.82
mlr2394	Chr	O	Molecular chaperone-GroEL	5.79
mll7465	Chr	G	ABC transporter permease	5.79
msr9689	pMLb	–	Hypothetical protein	5.62
msr8048	Chr	–	Hypothetical protein	5.61
msl2390	Chr	E	**Usg family protein**	5.54
msl1808	Chr	–	Hypothetical protein	5.49
mlr4836	Chr	HC	**Monooxygenase FAD-binding protein**	5.27
mlr2158	Chr	SR	**Metallo-beta-lactamase superfamily protein**	5.25
mlr2234	Chr	–	Hypothetical protein	5.25
mll9627	pMLb	O	sHSP	5.23
mlr2160	Chr	R	**Transporter component**	5.17
mlr5153	Chr	–	**Transmembrane protein**	5.09
mll9357	pMLa	S	**Domain-containing protein**	5.03
mll3694	Chr	T	Transcriptional regulator	4.98
mll1952	Chr	C	Norsolorinic acid reductase	4.98
mll4827	Chr	J	**Endoribonuclease L-PSP**	4.89
mlr2159	Chr	K	Transcriptional regulator	4.86
msr8615	Chr	R	**Transporter component**	4.82
msl3831	Chr	–	**Conserved hypothetical transmembrane protein**	4.79
mlr9581	pMLb	–	**PRC-barrel protein**	4.78
mll4607	Chr	S	**Ku protein**	4.66
mll1528	Chr	S	**Small integral membrane protein**	4.65
mlr8230	Chr	K	Transcriptional regulator	4.56
mlr0408	Chr	K	**Transmembrane anti-sigma factor**	4.56
msr2497	Chr	S	Hypothetical protein	4.47
mll8293	Chr	–	Hypothetical protein	4.38
msl2212	Chr	K	**Family transcriptional regulator**	4.36
msl7604	Chr	–	Hypothetical protein	4.31
msl7943	Chr	–	Hypothetical protein	4.28
msl9358	pMLa	S	**Transcription factor**	4.27
mlr2125	Chr	–	Hypothetical protein	4.27
mlr3707	Chr	–	Hypothetical protein	4.25
mlr0407	Chr	K	RNA polymerase sigma factor	4.16
mll3692	Chr	–	Hypothetical protein	4.14
msr8675	Chr	S	Hypothetical protein	4.11
mlr3233	Chr	N	**Host attachment protein**	4.07
msr4317	Chr	–	Hypothetical protein	4.04
mll2066	Chr	D	**Mobile mystery protein b**	4.02
mll6953	Chr	R	**Domain-containing protein**	4.02
mll6858	Chr	RIQ	Short chain dehydrogenase	3.98
mlr1797	Chr	S	**Conserved domain protein**	3.91
mll3445	Chr	C	**Luciferase-like protein**	3.91
mll2211	Chr	C	Morphinone reductase	3.89
msl6857	Chr	R	Hypothetical protein	3.88
mll8179	Chr	S	**Family protein**	3.83

The 50 genes with the highest *M*-values are shown. Gene descriptions shown in bold resulted from sequence analysis using Blast2GO software.

^a^COG category letters according to NCBI functional categories (http://www.ncbi.nlm.nih.gov/COG/grace/fiew.cgi).

Rhizobia genomes carry a large number of sHSP.^25^ Strain MAFF303099 has eight genes identified as sHSP, from which four were highly induced by heat shock (mll2387, mll2386, mll9627 and mll3033), two remained unaltered (mll2257 and mlr3192) and two were underexpressed (mlr4720 and mlr4721). sHSP can be divided into two classes in terms of sequence: class A includes sHSP similar to *E. coli* IbpAB, while sHSP grouped in class B are more divergent in terms of sequence.^25^ Gene mll2387 belongs to class A, while mll2386 is more divergent and considered a class B sHSP.^57^ According to Studer & Narberhaus^58^ it is improbable that mll2386 and mll2387 could form hetero-oligomers even if co-expressed, since in *B. japonicum* hetero-oligomers only occurred between sHSP from the same class. All class A sHSP from *M. loti* MAFF303099 (mll2387, mll3033, mlr3192 and mll9627-plasmid encoded) showed a ROSE element downstream to the promoter, which would confer high-temperature sensitivity to the transcription of these genes^26^ (Supplementary Fig. S2A). However, one of these sHSP was not overexpressed following the heat shock tested (mll3192-*hspH*), despite the fact that its *B. japonicum* homolog, also regulated by a ROSE element, is heat inducible.^25^

### 3.3. GroESL chaperone system

Similar to other heat shock related genes, rhizobia genomes harbour several copies of the *groESL* operon, usually with different regulation mechanisms and expression kinetics.^15^ *M. loti* MAFF303099 has four *groESL* operons in the chromosome and one in pMLa. From these five operons, only one appears to be involved in heat shock response, namely the *groEL* gene mlr2394, which was strongly overexpressed after heat shock exposure (*M* = 5.79). This *groEL* gene is highly similar to *groEL5* and *groEL1* from *S. meliloti* (87 and 83% amino acid identity, respectively), which are the most heat shock-inducible copies in that species.^5,27^

From what is known from other rhizobia genera, only some *groESL* operons encoded in the same genome are heat inducible and those can be regulated either by the σ^32^ or by CIRCE element.^12,13,59^ In the case of MAFF303099, a CIRCE element was found upstream all *groESL* operons (Supplementary Fig. S2B). The same exact consensus sequence of this inverted repeat is found in three operons and the remaining two operons differ in only two positions. The overexpressed *groEL* gene belongs to one of the operons regulated by a slightly divergent CIRCE element. Our results suggest that the presence of a CIRCE consensus sequence does not ensure a highly efficient induction under heat stress conditions. A similar situation was detected in *R. leguminosarum*, where a putative CIRCE element was found upstream of all three *groESL* operons, and further analysis of this regulation mechanism showed that the most heat-inducible operon was indeed CIRCE regulated, but a second operon, less induced by heat, was not affected by CIRCE deletion or *hrcA* knockout.^17^ This second operon was *rpoH* regulated, suggesting an overlapping of regulation mechanisms.^17^

Despite the high *M*-value detected for the *groEL* mlr2394, the expression of the *groES* gene in the same operon (mlr2393) following the heat shock remained unaltered. Similarly, in *S. meliloti*, the gene SMb22023 (*groES5*) was not induced by heat shock, despite the high induction of the corresponding *groEL5* gene (SMb21566).^5,27^ No promoter could be identified in the 59 bp *groES*–*groEL* intergenic space using BProm, so a bicistronic mRNA should be synthesized. A posttranscriptional cleavage could explain why only the transcript of the second gene in the operon is highly abundant. A cleavage event occurs in the *groESL* transcript of *Agrobacterium tumefaciens*, explaining why the transcript corresponding to *groEL* alone is the abundant mRNA detected after heat shock.^60^ Analysing the intergenic space in the MAFF303099 *groES*–*groEL* operon, using the ‘KineFold Web Server’,^61^ a stem-loop structure was found, though weaker than the one described to undergo cleavage in *A. tumefaciens* (data not shown). GroES–GroEL complexes comprising proteins encoded by different operons tend to be less efficient than the chaperonins complexes encoded by the same operon.^62^ However, the predominant GroES–GroEL complex consists of a single 10 kDa-heptameric ring (GroES) plus two rings of seven 60 kDa-monomers (GroEL), so the ratio between the two is 1:2, which is consistent with a lower *groES* transcription.

### 3.4. DnaKJ chaperone system

The role of the DnaKJ chaperone system in stress response is well known in other bacteria; however, few studies address these heat shock proteins in rhizobia. In the present study, *dnaK* (mll4757) and the co-chaperone *dnaJ* (mll4755) were not found to be significantly heat shock induced. Nevertheless, the real-time qRT–PCR results (Table 1) show that *dnaK* was induced by heat shock, agreeing with previous studies in *Mesorhizobium*.^16^ Approximately, 2-fold induction of the *dnaK* gene was detected in *S. meliloti* cells exposed to 40°C for 30 min,^5^ while no induction was reported for a shorter heat shock (42°C for 15 min).^27^ In the microarray analysis, the changes in expression levels of the *dnaK* gene were considered not statistically significant due to discrepancies among replicates.

It was reported for several rhizobia species that *dnaJ* deletions cause reduced growth at high temperatures;^21,24^ however, no transcriptional activation following a heat shock was detected in the present study or in other studies with *S. meliloti*.^5,27^ Similar to our results, no induction of *grpE* was reported for *S. meliloti* by Sauviac and coworkers,^27^ while a different study showed induction of *grpE* by heat shock.^5^

Another heat shock protein that has a close interaction with the DnaKJ system is ClpB. The *clpB* gene (mll3429) was found to be overexpressed in the present study, with an *M*-value of 2.93. The *clpB* gene was already seen to be upregulated following a heat shock in *S. meliloti*^5,27^ and the importance of ClpB in rhizobia stress response, especially to heat shock, was also previously reported.^63^ Similar to *E. coli*, the knockout of the *clpB* gene in *Mesorhizobium ciceri* led to an inability to endure high temperatures. Furthermore, in *M. ciceri* the symbiotic performance was also negatively affected.^63,64^ These results are consistent with the ClpB role in denatured protein disaggregation, namely by its cooperation with the DnaKJ system.^65^

### 3.5. Sigma factors

Rhizobia usually have multiple copies of genes encoding the same sigma factors, for example *rpoH* and *rpoE*. The *M. loti* MAFF303099 genome includes 25 putative sigma factors, from which four were induced by heat shock (mlr0407, mll3697, mll8140 and mlr3807). None of these sigma factor-encoding genes is completely annotated; nevertheless, BLAST analysis showed that loci mlr0407 (highly induced) and mll8140 are similar to both σ^70^ and σ^24^, and mlr3807 is more similar to σ^24^, while mll3697 shows high similarity to the *S. meliloti rpoE2* gene (76%). Sauviac and collaborators^27^ suggested RpoE2 as the major global regulator of stress response in *S. meliloti*, despite the fact that no phenotype change was detected in the *rpoE2* mutant. Our results are consistent with that suggestion, since mll3697 is overexpressed in heat shock conditions with an *M*-value of 2.4. The gene mll2869 encoding σ^70^ was found to be underexpressed following heat shock conditions, which may contribute to the extensive downregulation detected in MAFF303099 transcriptional response.

Sigma factors typically related to the heat shock response, as σ^32^ (*rpoH*) and σ^24^ (*rpoE*) that probably are encoded by mlr3741 and mlr8088 in MAFF303099, were not affected at the transcriptional level by the heat shock conditions applied. The gene *rpoH2* (mlr3862) was also not induced in the conditions used in this study. Similarly, Martínez-Salazar and coworkers^6^ reported that none of the *rpoH* genes were induced by heat shock in *R. etli*. Nevertheless, *rpoH* mutants are usually impaired in their stress tolerance phenotype, as is the case for *S. meliloti* and *R. etli*.^6,66^ *rpoH1* controls the expression of ∼21% of the heat shock-induced genes in *S. meliloti* and is also related to oxidative stress response, while *rpoH2* seems to play a minor role in the heat shock response and is more involved in osmotic tolerance.^5,6^

In *E. coli*, the *rpoH* regulation seems to be more at the protein level than at the transcriptional level. This control hypothesis is known as the ‘unfolded protein titration model’ and involves the most important chaperone systems: under normal growth conditions, σ^32^ binds to DnaKJ and GroESL so it becomes unavailable for RNA polymerase binding; under heat stress, misfolded proteins have higher affinity for chaperone systems and σ^32^ would be released.^67^ This posttranslational regulation has not been investigated in rhizobia, nevertheless the fact that no *rpoH* induction was detected under heat stress conditions is consistent with the proposed model.

### 3.6. Nodulation and nitrogen fixation genes

Some of the genes involved in nodulation and nitrogen fixation were detected to be differentially expressed after heat shock. Several *fix* genes showed severe underexpression, especially *fixK*, which encodes a transcriptional regulator and was the most underexpressed gene following the heat stress (Supplementary Table S2). The FixK is an activator for several operons, namely *fiXNOQP* and *fixGHIS* and the *fixK* gene is upregulated by micro-oxic conditions.^68^ MAFF303099 genome encodes two *fixNOPQ* operons (encoding cytochrome oxidases), one located in the symbiosis island. Interestingly, all the *fix* genes found to be underexpressed (*fixK*, *fixJ*, *fixS*, *fixI*, *fixP*, *fixO*, *fixN*) are outside the symbiosis island. Uchiumi and collaborators^69^ suggested that rhizobia might have acquired a housekeeping *fixNOPQ* operon before the acquisition of the symbiosis island. Similar to the present study, *fix* genes were previously detected to be underexpressed after a heat shock in *S. meliloti*,^5^ so a downregulation of the *fixK* cascade upon high-temperature conditions seems to be consistent. In *S. meliloti*, *fixK* is negatively regulated by the activity of *fixT*; however, no *fixT* gene is annotated in MAFF303099 genome (the most similar gene is msl5852, which is not differentially expressed in the present study).

From the high number of nodulation genes encoded in the *M. loti* MAFF303099 genome (>40 genes) only 11 showed altered transcript levels after the heat shock. The genes *nodC* and *nodE* were heat induced, while nine other nodulation genes were underexpressed. Only *nodL* was previously reported to be underexpressed following heat shock conditions in *S. meliloti*^5,27^ but this gene expression remained unaltered in the present study.

### 3.7. Other heat shock-inducible genes

Among the 50 genes with the highest *M*-values (Table 2), there are five transcriptional regulators, one sigma factor and one anti-sigma factor, which indicates that heat shock response is a complex system with relevant control at the transcriptional level.

Additional analysis of all hypothetical proteins differentially expressed performed in this study, allowed further characterization of many genes, for example mll4607, which is now annotated as Ku protein (Table 2). Together with LigD this protein is involved in DNA repair, namely in the repair of non-homologous end-joining of double-strand DNA.^70^ Unlike other bacteria, rhizobial genomes encode multiple copies of this Ku/LigD system, which has been further studied in *S. meliloti*.^71^ Although none of the *ku* homologues is required for the symbiosis establishment, this DNA repair system is active in both free-living cells and bacteroids.^71^ From the four *ku* homologs in the MAFF303099 genome, three are induced by heat shock (mll4607, mlr9624 and mlr9623), as well as one of the three *ligD* homologues (mll9625). Until recently, double-stranded DNA breaks (DSB) were not thought to be a consequence of heat shock; however, a recent study, using eukaryotic cells, showed that heat shock may in fact induce DSB on certain phases of the cell cycle.^72^ It is tempting to agree with the suggestion from Kobayashi and coworkers^71^ that these systems do have some role under stress conditions, such as heat shock.

Altogether our results suggest that in a large bacterial genome, the extensive gene downregulation may be an important part of the heat shock response. Although the present study has contributed to further knowledge on rhizobia stress response, future studies are required to understand the role of individual genes and the mechanisms regulating these molecular responses.

## Supplementary data

Supplementary Data are available at www.dnaresearch.oxfordjournals.org.

## Funding

This work was funded by Fundação para a Ciência e a Tecnologia (FCT), including the research projects FCOMP-01-0124-FEDER-007091, PTDC/BIA-EVF/4158/2012 and the strategic project PEst-C/AGR/UI0115/2011, that include FEDER funds through the Operational Programme for Com-petitiveness Factors—COMPETE and National funds. A. A. and M. L. acknowledge postdoctoral fellowships from FCT (SFRH/BPD/73243/2010 and SFRH/BPD/27008/2006).

## Supplementary Material

Supplementary Data

## References

[1] Jensen E.S., Hauggaard-Nielsen H. (2003). How can increased use of biological N_2_ fixation in agriculture benefit the environment?. Plant and Soil.

[2] MacLean A.M., Finan T.M., Sadowsky M.J. (2007). Genomes of the symbiotic nitrogen-fixing bacteria of legumes. Plant Physiol..

[3] Richter K., Haslbeck M., Buchner J. (2010). The heat shock response: life on the verge of death. Mol. Cell.

[4] Gruber T.M., Gross C.A. (2003). Multiple sigma subunits and the partitioning of bacterial transcription space. Annu. Rev. Microbiol..

[5] Barnett M.J., Bittner A.N., Toman C.J., Oke V., Long S.R. (2012). Dual RpoH sigma factors and transcriptional plasticity in a symbiotic bacterium. J. Bacteriol..

[6] Martínez-Salazar J.M., Sandoval-Calderon M., Guo X.W. (2009). The *Rhizobium etli* RpoH1 and RpoH2 sigma factors are involved in different stress responses. Microbiology.

[7] Mitsui H., Sato T., Sato Y., Ito N., Minamisawa K. (2004). *Sinorhizobium meliloti* RpoH(1) is required for effective nitrogen-fixing symbiosis with alfalfa. Mol. Genet. Genomics.

[8] Hartl F.U., Hayer-Hartl M. (2009). Converging concepts of protein folding *in vitro* and *in vivo*. Nat. Struct. Mol. Biol..

[9] Ferrer M., Chernikova T.N., Yakimov M.M., Golyshin P.N., Timmis K.N. (2003). Chaperonins govern growth of *Escherichia coli* at low temperatures. Nat. Biotechnol..

[10] Kim S.Y., Ayyadurai N., Heo M.A., Park S., Jeong Y.J., Lee S.G. (2009). Improving the productivity of recombinant protein in *Escherichia coli* under thermal stress by coexpressing GroELS chaperone system. J. Microbiol. Biotechnol..

[11] Rudolph B., Gebendorfer K.M., Buchner J., Winter J. (2010). Evolution of *Escherichia coli* for growth at high temperatures. J. Biol. Chem..

[12] Bittner A.N., Foltz A., Oke V. (2007). Only one of five *groEL* genes is required for viability and successful symbiosis in *Sinorhizobium meliloti*. J. Bacteriol..

[13] Babst M., Hennecke H., Fischer H.M. (1996). Two different mechanisms are involved in the heat-shock regulation of chaperonin gene expression in *Bradyrhizobium japonicum*. Mol. Microbiol..

[14] Gould P.S., Burglar H.R., Lund P.A. (2007). Homologous cpn60 genes in *Rhizobium leguminosarum* are not functionally equivalent. Cell Stress Chaperon..

[15] Alexandre A., Oliveira S. (2013). Response to temperature stress in rhizobia. Crit. Rev. Microbiol..

[16] Alexandre A., Oliveira S. (2011). Most heat-tolerant rhizobia show high induction of major chaperone genes upon stress. FEMS Microbiol. Ecol..

[17] Gould P., Maguire M., Lund P.A. (2007). Distinct mechanisms regulate expression of the two major *groEL* homologues in *Rhizobium leguminosarum*. Arch. Microbiol..

[18] Bittner A.N., Oke V. (2006). Multiple *groESL* operons are not key targets of RpoH1 and RpoH2 in *Sinorhizobium meliloti*. J. Bacteriol..

[19] Zuber U., Schumann W. (1994). CIRCE, a novel heat-shock element involved in regulation of heat-shock operon *dnaK* of *Bacillus subtilis*. J. Bacteriol..

[20] Schulz A., Schumann W. (1996). *hrcA*, the first gene of the *Bacillus subtilis dnaK* operon encodes a negative regulator of class I heat shock genes. J. Bacteriol..

[21] Minder A.C., Narberhaus F., Babst M., Hennecke H., Fischer H.M. (1997). The *dnaKJ* operon belongs to the sigma(32)-dependent class of heat shock genes in *Bradyrhizobium japonicum*. Mol. Gen. Genet..

[22] Labidi M., Laberge S., Vezina L.P., Antoun H. (2000). The *dnaJ* (hsp40) locus in *Rhizobium leguminosarum* bv. *phaseoli* is required for the establishment of an effective symbiosis with *Phaseolus vulgaris*. Mol. Plant-Microbe Interact..

[23] Gomes D.F., da Silva Batista J.S., Schiavon A.L., Andrade D.S., Hungria M. (2012). Proteomic profiling of *Rhizobium tropici* PRF 81: identification of conserved and specific responses to heat stress. BMC Microbiol..

[24] Nogales J., Campos R., BenAbdelkhalek H., Olivares J., Lluch C., Sanjuan J. (2002). *Rhizobium tropici* genes involved in free-living salt tolerance are required for the establishment of efficient nitrogen-fixing symbiosis with *Phaseolus vulgaris*. Mol. Plant-Microbe Interact..

[25] Münchbach M., Nocker A., Narberhaus F. (1999). Multiple small heat shock proteins in rhizobia. J. Bacteriol..

[26] Narberhaus F., Waldminighaus T., Chowdhury S. (2006). RNA Thermometers. FEMS Microbiol. Rev..

[27] Sauviac L., Philippe H., Phok K., Bruand C. (2007). An extracytoplasmic function sigma factor acts as a general stress response regulator in *Sinorhizobium meliloti*. J. Bacteriol..

[28] Kaneko T., Nakamura Y., Sato S. (2000). Complete genome structure of the nitrogen-fixing symbiotic bacterium *Mesorhizobium loti*. DNA Res..

[29] Laranjo M., Alexandre A., Oliveira S. Legume growth-promoting rhizobia: an overview on the *Mesorhizobium* genus. Microbiol. Res.

[30] Laranjo M., Oliveira S. (2011). Tolerance of *Mesorhizobium* type strains to different environmental stresses. Antonie van Leeuwenhoek.

[31] Vincent J.M. (1970). A Manual for the Practical Study of Root-Nodule Bacteria.

[32] Simon R., Lam A., Li M.-C., Ngan M., Menenzes S., Zhao Y. (2007). Analysis of gene expression data using BRB-ArrayTools. Cancer Inform..

[33] Saeed A.I., Hagabati N.K., Braisted J.C. (2006). TM4 microarray software suite. DNA Microarrays B Databases Stat..

[34] Gotz S., Garcia-Gomez J.M., Terol J. (2008). High-throughput functional annotation and data mining with the Blast2GO suite. Nucleic Acids Res..

[35] Szklarczyk D., Franceschini A., Kuhn M. (2011). The STRING database in 2011: functional interaction networks of proteins, globally integrated and scored. Nucleic Acids Res..

[36] Price M.N., Huang K.H., Alm E.J., Arkin A.P. (2005). A novel method for accurate operon predictions in all sequenced prokaryotes. Nucleic Acids Res..

[37] Carver T., Thomson N., Bleasby A., Berriman M., Parkhill J. (2009). DNAPlotter: circular and linear interactive genome visualization. Bioinformatics.

[38] Madsen M.L., Nettleton D., Thacker E.L., Edwards R., Minion F.C. (2006). Transcriptional profiling of *Mycoplasma hyopneumoniae* during heat shock using microarrays. Infect. Immun..

[39] Crapoulet N., Barbry P., Raoult D., Renesto P. (2006). Global transcriptome analysis of *Tropheryma whipplei* in response to temperature stresses. J. Bacteriol..

[40] Audia J.P., Patton M.C., Winkler H.H. (2008). DNA microarray analysis of the heat shock transcriptome of the obligate intracytoplasmic pathogen *Rickettsia prowazekii*. Appl. Environ. Microbiol..

[41] Stintzi A. (2003). Gene expression profile of *Campylobacter jejuni* in response to growth temperature variation. J. Bacteriol..

[42] Li J.-s., Bi Y.-t., Dong C., Yang J.-f., Liang W.-d. (2011). Transcriptome analysis of adaptive heat shock response of *Streptococcus thermophilus*. PLoS ONE.

[43] Rohlin L., Trent J.D., Salmon K., Kim U., Gunsalus R.P., Liao J.C. (2005). Heat shock response of *Archaeoglobus fulgidus*. J. Bacteriol..

[44] Rezzonico E., Lariani S., Barretto C. (2007). Global transcriptome analysis of the heat shock response of *Bifidobacterium longum*. FEMS Microbiol. Lett..

[45] Koide T., Vencio R.Z.N., Gomes S.L. (2006). Global gene expression analysis of the heat shock response in the phytopathogen *Xylella fastidiosa*. J. Bacteriol..

[46] van der Veen S., Hain T., Wouters J.A. (2007). The heat-shock response of *Listeria monocytogenes* comprises genes involved in heat shock. cell division, cell wall synthesis, and the SOS response, *Microbiology*.

[47] Yin H., Tang M., Zhou Z. (2012). Distinctive heat-shock response of bioleaching microorganism *Acidithio-bacillus ferrooxidans* observed using genome-wide microarray. Can. J. Microbiol..

[48] Barreiro C., Nakunst D., Hueser A.T., de Paz H.D., Kalinowski J., Martin J.F. (2009). Microarray studies reveal a ‘differential response’ to moderate or severe heat shock of the HrcA- and HspR-dependent systems in *Corynebacterium glutamicum*. Microbiology.

[49] Chhabra S.R., He Q., Huang K.H. (2006). Global analysis of heat shock response in *Desulfovibrio vulgaris* Hildenborough. J. Bacteriol..

[50] Emerson J.E., Stabler R.A., Wren B.W., Fairweather N.F. (2008). Microarray analysis of the transcriptional responses of *Clostridium difficile* to environmental and antibiotic stress. J. Med. Microbiol..

[51] Richmond C.S., Glasner J.D., Mau R., Jin H.F., Blattner F.R. (1999). Genome-wide expression profiling in *Escherichia coli* K12. Nucleic Acids Res..

[52] Zhang W., Culley D.E., Nie L., Brockman F.J. (2006). DNA microarray analysis of anaerobic *Methanosarcina barkeri* reveals responses to heat shock and air exposure. J. Ind. Microbiol. Biotechnol..

[53] Gao H.C., Wang Y., Liu X.D. (2004). Global transcriptome analysis of the heat shock response of *Shewanella oneidensis*. J. Bacteriol..

[54] Wren J.D., Conway T. (2006). Meta-analysis of published transcriptional and translational fold changes reveals a preference for low-fold inductions. OMICS.

[55] Koonin E.V., McEntyre J., Ostell J. (2002). The Clusters of Orthologous Groups (COGs) Database: Phylogenetic Classification of Proteins from Complete Genomes. The NCBI Handbook.

[56] Konstantinidis K.T., Tiedje J.M. (2004). Trends between gene content and genome size in prokaryotic species with larger genomes. Proc. Natl. Acad. Sci. USA.

[57] Nocker A., Krstulovic N.P., Perret X., Narberhaus F. (2001). ROSE elements occur in disparate rhizobia and are functionally interchangeable between species. Arch. Microbiol..

[58] Studer S., Narberhaus F. (2000). Chaperone activity and homo- and hetero-oligomer formation of bacterial small heat shock proteins. J. Biol. Chem..

[59] Rodriguez-Quinones F., Maguire M., Wallington E.J. (2005). Two of the three *groEL* homologues in *Rhizobium leguminosarum* are dispensable for normal growth. Arch. Microbiol..

[60] Segal G., Ron E.Z. (1995). The *dna*KJ operon of *Agrobacterium tumefaciens*: transcriptional analysis and evidence for a new heat shock promoter. J. Bacteriol..

[61] Xayaphoummine A., Bucher T., Isambert H. (2005). Kinefold web server for RNA/DNA folding path and structure prediction including pseudoknots and knots. Nucleic Acids Res..

[62] Wang J.D., Herman C., Tipton K.A., Gross C.A., Weissman J.S. (2002). Directed evolution of substrate-optimized GroEL/S chaperonins. Cell.

[63] Brígido C., Robledo M., Menendez E., Mateos P.F., Oliveira S. (2012). A ClpB chaperone knockout mutant of *Mesorhizobium ciceri* shows a delay in the root nodulation of chickpea plants. Mol. Plant-Microbe Interact..

[64] Squires C.L., Pedersen S., Ross B.M., Squires C. (1991). ClpB is the *Escherichia coli* heat-shock protein F84.1. J. Bacteriol..

[65] Seyffer F., Kummer E., Oguchi Y. (2012). Hsp70 proteins bind Hsp100 regulatory M domains to activate AAA plus disaggregase at aggregate surfaces. Nat. Struct. Mol. Biol..

[66] Ono Y., Mitsui H., Sato T., Minamisawa K. (2001). Two RpoH homologs responsible for the expression of heat shock protein genes in *Sinorhizobium meliloti*. Mol. Gen. Genet..

[67] Guisbert E., Yura T., Rhodius V.A., Gross C.A. (2008). Convergence of molecular, modeling, and systems approaches for an understanding of the *Escherichia coli* heat shock response. Microbiol. Mol. Biol. Rev..

[68] Dixon R., Kahn D. (2004). Genetic regulation of biological nitrogen fixation. Nat. Rev. Microbiol..

[69] Uchiumi T., Ohwada T., Itakura M. (2004). Expression islands clustered on the symbiosis island of the *Mesorhizobium loti* genome. J. Bacteriol..

[70] Gong C.L., Bongiorno P., Martins A. (2005). Mechanism of nonhomologous end-joining in mycobacteria: a low-fidelity repair system driven by Ku, ligase D and ligase C. Nat. Struct. Mol. Biol..

[71] Kobayashi H., Simmons L.A., Yuan D.S., Broughton W.J., Walker G.C. (2008). Multiple Ku orthologues mediate DNA non-homologous end-joining in the free-living form and during chronic infection of *Sinorhizobium meliloti*. Mol. Microbiol..

[72] Velichko A.K., Petrova N.V., Kantidze O.L., Razin S.V. (2012). Dual effect of heat shock on DNA replication and genome integrity. Mol. Biol. Cell.

