# Supplementary material for: Global Transcriptional Response to Heat Shock of the Legume Symbiont Mesorhizobium loti MAFF303099 Comprises Extensive Gene Downregulation
Source: DNA Res. 2013 Nov 25;21(2):195–206. doi: 10.1093/dnares/dst050 (PMC3989490; doi:10.1093/dnares/dst050)
Supplement: Supplementary Data [file supp_21_2_195__index.html]

Global Transcriptional Response to Heat Shock of the Legume Symbiont Mesorhizobium loti MAFF303099 Comprises Extensive Gene Downregulation — Global Transcriptional Response to Heat Shock of the Legume Symbiont Mesorhizobium loti MAFF303099 Comprises Extensive Gene Downregulation — Supplementary Data 

# Global Transcriptional Response to Heat Shock of the Legume Symbiont *Mesorhizobium loti* MAFF303099 Comprises Extensive Gene Downregulation

## Supplementary Data

Supplementary Data

**Files in this Data Supplement:**

- Supplementary Figure 1 - ppt file
- Supplementary Figure 2 - tif file
- Supplementary Table 1 - doc file
- Supplementary Table 2 - doc file
