# Supplementary figures and images for: Global Transcriptional Response to Heat Shock of the Legume Symbiont Mesorhizobium loti MAFF303099 Comprises Extensive Gene Downregulation
Source: DNA Res. 2013 Nov 25;21(2):195–206. doi: 10.1093/dnares/dst050 (PMC3989490; doi:10.1093/dnares/dst050)

## Slide 1
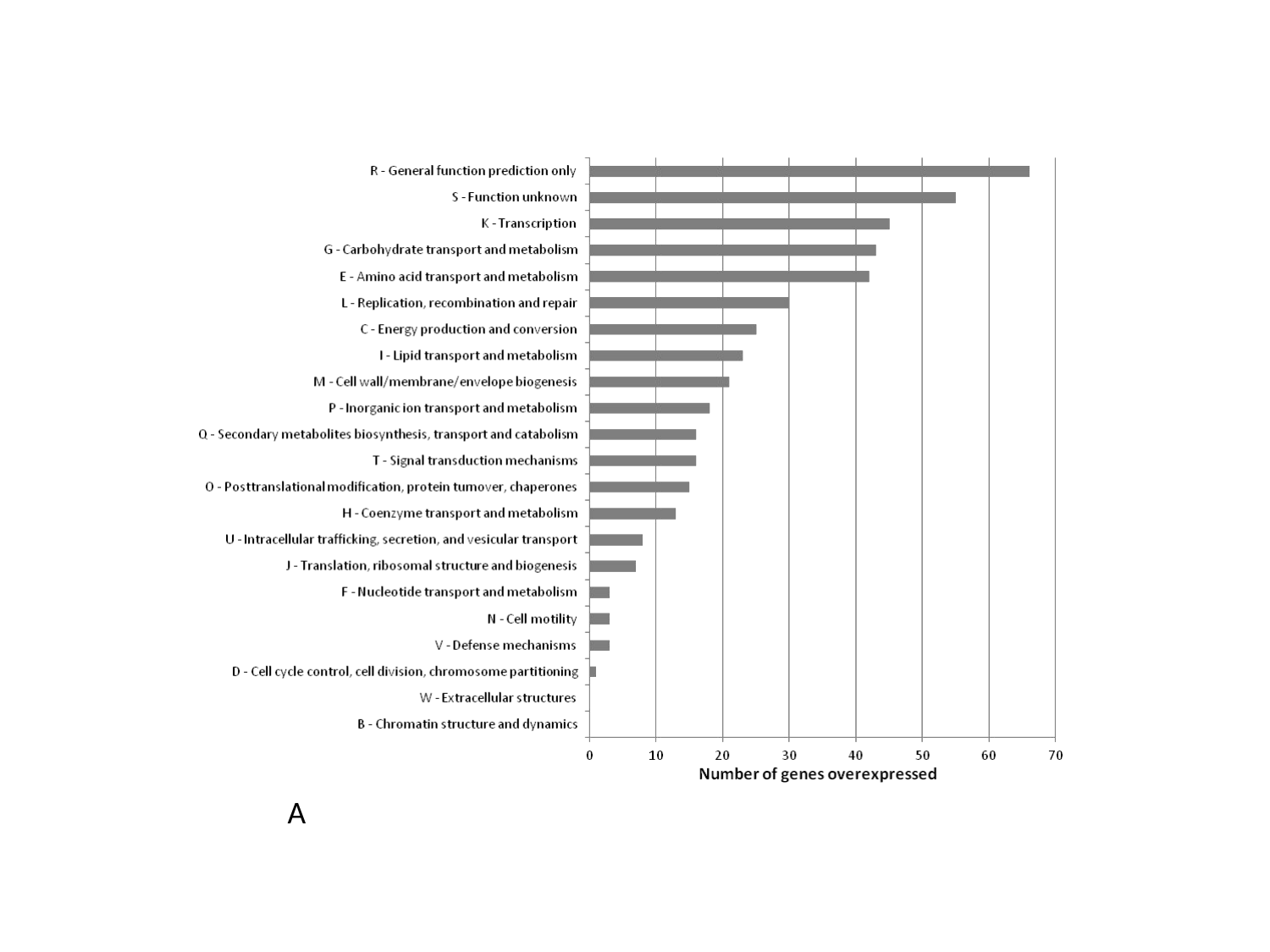

A

## Slide 2
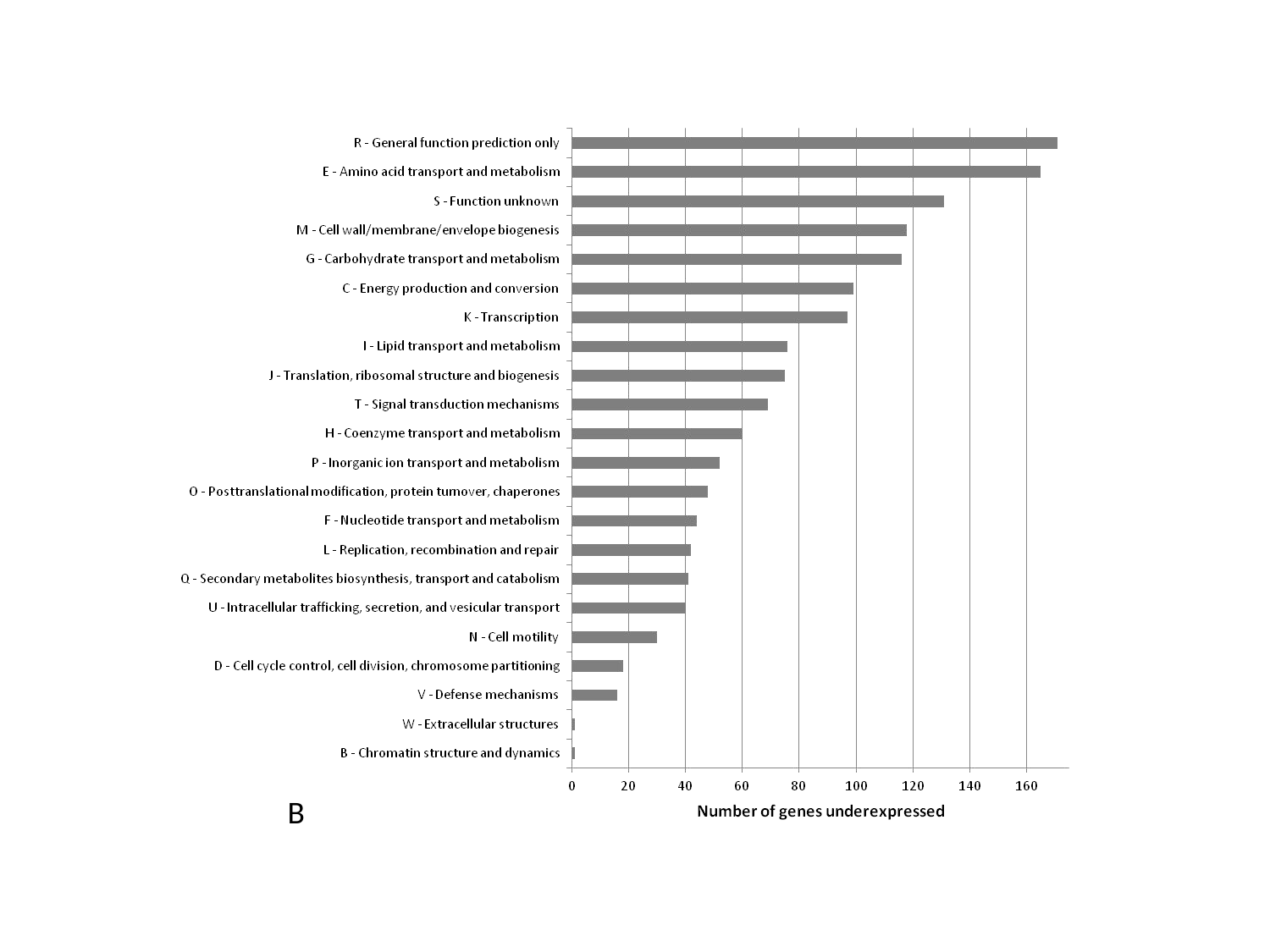

B

Supplement: Supplementary Data [file supp_dst050_dst050supp_fig1.ppt]

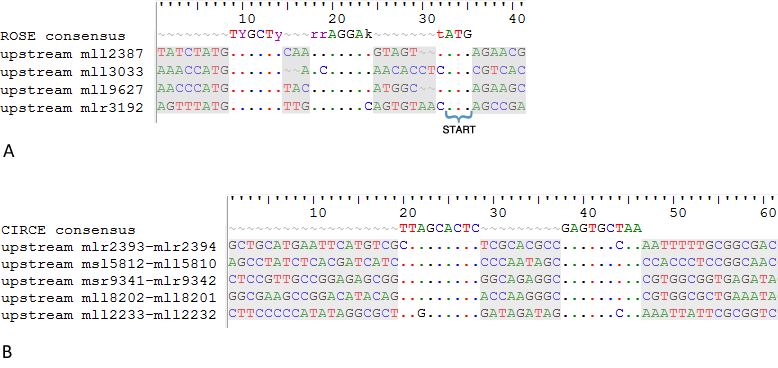

Supplement: Supplementary Data [file supp_dst050_dst050supp_fig2.tif]
