# Supplementary material for: Global Transcriptional Response to Heat Shock of the Legume Symbiont Mesorhizobium loti MAFF303099 Comprises Extensive Gene Downregulation
Source: DNA Res. 2013 Nov 25;21(2):195–206. doi: 10.1093/dnares/dst050 (PMC3989490; doi:10.1093/dnares/dst050)
Supplement: Supplementary Data [file supp_dst050_dst050supp_table1.doc]

Table S1 – List of primers used for real time qRT-PCR.

| Locus tag | Forward primer | Reverse primer |
| --- | --- | --- |
| mll2386 | 5’-CGCGGTGACGACATGGA-3’ | 5’-TCGTCGAATAGCCGGTTGA-3’ |
| mlr2394 | 5’-GTCGTAGAGGGCATGCAATTC-3’ | 5’-GACGCGCATCTTGTCCTGAT-3’ |
| mll1528 | 5’-TCACCAGGATCGCCAATTG-3’ | 5’-AGCAGCCGGCGAATGTC-3’ |
| mll3429 | 5’-GGAGCTTGTCGGCCTTGA-3’ | 5’-AAGCCCGAGCTTCTGCTTCT-3’ |
| mll3842 | 5’-AAAAGCGCTCGACACCTATCTG-3’ | 5’-CGAAGGTCGAGGCGTTGA-3’ |
| mlr5932 | 5’-GTTTTTTTCCAGCGGGCTC-3’ | 5’-TTGATGGCCGGCGC-3’ |
| mll3873 | 5’-GCTGTCCATCATCGGCAAT-3’ | 5’-GCCTTGAGCTTCGCCACTT-3’ |
| mlr0883 | 5’-ACGGGCGACGACTCAAAAC-3’ | 5’-ATCGACCAACCGGCAAAG-3’ |
| mlr6118 | 5’-GCGAGGGCACGGTCAA-3’ | 5’-CCGTTTTCAGGAGCAATGAAG-3’ |
| mll1546 | 5’-CAAGCCGTTCCACTTCGAA-3’ | 5’-CGAAGGTCGTTTTGTCATTGG-3’ |
| mll6630 | 5’-GAGCCTTTCCGACAGCATATGT-3’ | 5’-ACTTGTGTTGCGCAGAAGAACA3’ |
| mlr2911 | 5’-GGAGCCTGTTTCCCTTTTCG-3’ | 5’-GCGCACGGAAAGCCATT-3’ |
| mll6578 | 5’-TCGTTGCCGTCGCATTCT-3’ | 5’-GTTGCGTTTCCGAGTCGAA-3’ |
| mll6432 | 5’-TTCCGACAACACGCTTAACG-3’ | 5’-CGCGGTAGCTTCTTCTTTGC-3’ |
| mll4757 | 5’-GGCATCGACCTGAAGAACGA-3’ | 5’-TGAAGGGCAGGTTGATTTCG-3’ |
| mll4755 | 5’-CCGACGAGAAGGAGCTCAAG-3’ | 5’-TGCGGTCAGGATGGAACTG-3’ |
| mlr7618 | 5’-AAGAAGGTGCATTGGAAAATCG-3’ | 5’-GCGAAACATGCGAAATGCTT-3’ |
