# Supplementary material for: Global Transcriptional Response to Heat Shock of the Legume Symbiont Mesorhizobium loti MAFF303099 Comprises Extensive Gene Downregulation
Source: DNA Res. 2013 Nov 25;21(2):195–206. doi: 10.1093/dnares/dst050 (PMC3989490; doi:10.1093/dnares/dst050)
Supplement: Supplementary Data [file supp_dst050_dst050supp_table2.doc]

Table S2 - Differentially expressed genes in response to heat shock for *Mesorhizobium* *loti* MAFF303099.

| **Locus tag** | **M-values** |
| --- | --- |
| mll2386 | 6,606254 |
| mll2387 | 6,321996 |
| mll3685 | 6,078668 |
| msl2054 | 5,840168 |
| mll1959 | 5,822225 |
| mlr2394 | 5,794425 |
| mll7465 | 5,785266 |
| msr9689 | 5,620732 |
| msr8048 | 5,606389 |
| msl2390 | 5,538298 |
| msl1808 | 5,488146 |
| mlr4836 | 5,2674495 |
| mlr2158 | 5,245703 |
| mlr2234 | 5,245452 |
| mll9627 | 5,22619 |
| mlr2160 | 5,172305 |
| mlr5153 | 5,092017 |
| mll9357 | 5,028638 |
| mll3694 | 4,978108 |
| mll1952 | 4,975902 |
| mll4827 | 4,888813 |
| mlr2159 | 4,858466 |
| msr8615 | 4,822753 |
| msl3831 | 4,790658 |
| mlr9581 | 4,77671 |
| mll4607 | 4,656447 |
| mll1528 | 4,652101 |
| mlr8230 | 4,5636095 |
| mlr0408 | 4,562929 |
| msr2497 | 4,465158 |
| mll8293 | 4,378876 |
| msl2212 | 4,361869 |
| msl7604 | 4,307477 |
| msl7943 | 4,279126 |
| msl9358 | 4,274109 |
| mlr2125 | 4,271948 |
| mlr3707 | 4,2520035 |
| mlr0407 | 4,159789 |
| mll3692 | 4,142024 |
| msr8675 | 4,112526 |
| mlr3233 | 4,073726 |
| msr4317 | 4,043543 |
| mll2066 | 4,017225 |
| mll6953 | 4,016239 |
| mll6858 | 3,982618 |
| mlr1797 | 3,907981 |
| mll3445 | 3,905232 |
| mll2211 | 3,89227 |
| msl6857 | 3,875439 |
| mll8179 | 3,832437 |
| mll9648 | 3,830886 |
| mll4599 | 3,775308 |
| mll1975 | 3,7693065 |
| mlr4318 | 3,726449 |
| msl5664 | 3,676041 |
| mll0693 | 3,671054 |
| mlr8082 | 3,666435 |
| mll9638 | 3,662552 |
| mlr1472 | 3,647633 |
| mll2392 | 3,630774 |
| mlr1777 | 3,621401 |
| mlr7815 | 3,5859565 |
| mll3139 | 3,580442 |
| mll2068 | 3,521086 |
| mll9633 | 3,507065 |
| msr0731 | 3,503013 |
| mlr9752 | 3,497318 |
| msr0529 | 3,4566745 |
| mll0592 | 3,447756 |
| mlr1783 | 3,433517 |
| mlr2819 | 3,416523 |
| mll0867 | 3,4115 |
| mlr3704 | 3,410452 |
| mlr2832 | 3,382374 |
| mll4093 | 3,37334 |
| msr7945 | 3,369673 |
| msl6954 | 3,344274 |
| mll4816 | 3,328356 |
| msr9531 | 3,3274495 |
| mll2215 | 3,327084 |
| msr7955 | 3,320105 |
| mll9651 | 3,300796 |
| mll3568 | 3,262455 |
| mll2378 | 3,258692 |
| msr0083 | 3,255359 |
| mll2592 | 3,252681 |
| mll5835 | 3,240809 |
| mll9637 | 3,240099 |
| mlr4819 | 3,235056 |
| mll4092 | 3,22664 |
| msl0931 | 3,222307 |
| msr2235 | 3,211796 |
| mll2216 | 3,189448 |
| mlr3102 | 3,189115 |
| mll4358 | 3,156136 |
| mll9685 | 3,155836 |
| msl7942 | 3,1558225 |
| mlr2526 | 3,151104 |
| mlr3686 | 3,146506 |
| msr9764 | 3,14113 |
| mlr0409 | 3,134247 |
| msl3727 | 3,118181 |
| mll0933 | 3,076236 |
| mlr8231 | 3,07584 |
| mll4764 | 3,0724975 |
| mll4619 | 3,069698 |
| msr9666 | 3,062133 |
| mlr3700 | 3,056888 |
| mlr3964 | 3,042621 |
| mll8064 | 3,041615 |
| msl3142 | 3,03946 |
| mlr1652 | 3,039356 |
| mlr3136 | 3,009622 |
| msl1572 | 2,993037 |
| mlr9679 | 2,990591 |
| mlr9687 | 2,979438 |
| mll8063 | 2,97377 |
| mlr9533 | 2,937389 |
| mll4821 | 2,935745 |
| mll4820 | 2,928846 |
| mll3447 | 2,928379 |
| mlr3020 | 2,928089 |
| mll3429 | 2,92137 |
| msr8560 | 2,904869 |
| mll2299 | 2,832623 |
| mlr2854 | 2,832618 |
| mll9650 | 2,8014495 |
| mlr5173 | 2,77563 |
| mll6995 | 2,77342 |
| msl6994 | 2,763677 |
| msr8120 | 2,761695 |
| mlr6370 | 2,758744 |
| mlr9549 | 2,729846 |
| msl3295 | 2,715373 |
| msl3143 | 2,706473 |
| msr8122 | 2,704474 |
| mlr9624 | 2,691555 |
| mll2276 | 2,6902685 |
| msr2059 | 2,689216 |
| mlr9579 | 2,689021 |
| mlr2029 | 2,686815 |
| mlr1858 | 2,681106 |
| msr8113 | 2,674133 |
| mll2118 | 2,664513 |
| mlr5278 | 2,658603 |
| mll1513 | 2,657783 |
| mlr4033 | 2,632316 |
| mll7603 | 2,617541 |
| mlr2855 | 2,612305 |
| mll4533 | 2,598085 |
| mlr9078 | 2,589655 |
| msl8039 | 2,58256 |
| mlr2375 | 2,56543 |
| mll3444 | 2,5611795 |
| mlr1748 | 2,55651 |
| mll9683 | 2,543826 |
| mll3492 | 2,531486 |
| mll9625 | 2,530659 |
| mll0691 | 2,530176 |
| msr2723 | 2,507093 |
| mlr8233 | 2,498419 |
| mll6136 | 2,498313 |
| mll7714 | 2,492786 |
| mll7977 | 2,484015 |
| msr9628 | 2,481953 |
| mll2589 | 2,465728 |
| msr7956 | 2,452868 |
| msl1788 | 2,449135 |
| msr9566 | 2,441877 |
| mll1760 | 2,416108 |
| mll3697 | 2,4154 |
| mlr9262 | 2,411642 |
| mll2385 | 2,405114 |
| mll3691 | 2,403508 |
| mlr9690 | 2,403096 |
| mlr2161 | 2,3980955 |
| mlr7958 | 2,378781 |
| mll5360 | 2,370556 |
| msl0434 | 2,36673 |
| mlr2535 | 2,353169 |
| mll2459 | 2,335443 |
| mlr2421 | 2,333416 |
| mlr9645 | 2,328897 |
| mlr4818 | 2,315673 |
| mlr5136 | 2,310708 |
| msr3706 | 2,294761 |
| msl1957 | 2,2896315 |
| mlr2831 | 2,266892 |
| mll2277 | 2,247737 |
| mlr3103 | 2,242505 |
| mll5358 | 2,2407395 |
| mll3842 | 2,238413 |
| mll3341 | 2,238103 |
| mlr7388 | 2,227432 |
| mll2618 | 2,214385 |
| msr3702 | 2,186862 |
| msr5733 | 2,185702 |
| mll1854 | 2,150975 |
| msr4805 | 2,128046 |
| mll8081 | 2,104235 |
| msl0431 | 2,089255 |
| mll3033 | 2,084554 |
| mll8059 | 2,0570185 |
| mlr9079 | 2,055682 |
| mll5898 | 2,050632 |
| msl7979 | 2,0438675 |
| mll2830 | 2,043076 |
| mlr0530 | 2,040951 |
| msl0430 | 2,031153 |
| msr3137 | 2,008456 |
| mlr2046 | 2,008166 |
| mll9356 | 1,9909695 |
| msr9526 | 1,9873585 |
| mlr0026 | 1,980551 |
| mll6233 | 1,953078 |
| mlr0239 | 1,92179 |
| mll5834 | 1,913297 |
| mll6889 | 1,899381 |
| msr7985 | 1,896415 |
| mlr5281 | 1,892523 |
| msr8429 | 1,880184 |
| msr2379 | 1,875595 |
| mll1856 | 1,869719 |
| msl9670 | 1,855732 |
| mlr5903 | 1,855579 |
| mlr8558 | 1,840216 |
| mll2784 | 1,836095 |
| mll0142 | 1,822341 |
| mll9629 | 1,810368 |
| msr7957 | 1,773104 |
| mll3374 | 1,772651 |
| mll0782 | 1,77189 |
| mll4705 | 1,749084 |
| mll5900 | 1,738515 |
| mll9691 | 1,669172 |
| mlr1780 | 1,645728 |
| mlr7924 | 1,5771345 |
| mlr1142 | 1,568093 |
| mlr9700 | 1,537667 |
| mlr0240 | 1,535858 |
| mlr0358 | 1,53315 |
| mlr9360 | 1,523624 |
| mll7029 | 1,455481 |
| mll9301 | 1,45068 |
| msr2057 | 1,450542 |
| msr0114 | 1,409944 |
| mlr5374 | 1,404915 |
| mll9055 | 1,400387 |
| mll6039 | 1,393219 |
| mlr9590 | 1,384121 |
| mll2507 | 1,380558 |
| mll2263 | 1,374126 |
| mll1963 | 1,370825 |
| mlr9748 | 1,368765 |
| mlr4517 | 1,359433 |
| mlr3054 | 1,354958 |
| msl6054 | 1,3464565 |
| mlr0237 | 1,344158 |
| mll3127 | 1,3399415 |
| mll0476 | 1,3370355 |
| mll5199 | 1,313668 |
| mlr5732 | 1,305466 |
| msl6419 | 1,293903 |
| mll6410 | 1,29251 |
| msr1111 | 1,2810215 |
| mll0781 | 1,2746105 |
| mll8140 | 1,269138 |
| mlr6955 | 1,244105 |
| mlr7407 | 1,238811 |
| mll6408 | 1,236254 |
| msl3283 | 1,235415 |
| mlr1700 | 1,234165 |
| mlr2588 | 1,211491 |
| mll2271 | 1,210582 |
| msr0834 | 1,200097 |
| mlr6754 | 1,196991 |
| mlr1828 | 1,1930305 |
| mll7085 | 1,186382 |
| mlr4527 | 1,182705 |
| mll5613 | 1,161639 |
| mlr5479 | 1,160655 |
| mlr8076 | 1,158832 |
| mlr8013 | 1,157048 |
| mlr4529 | 1,155503 |
| mlr6653 | 1,1515975 |
| mlr5040 | 1,145797 |
| mll8564 | 1,1399 |
| mlr5932 | 1,1379985 |
| mll8760 | 1,137757 |
| mll1508 | 1,1374855 |
| mlr4191 | 1,128573 |
| mlr2187 | 1,128231 |
| msr9519 | 1,1270805 |
| msr8167 | 1,124694 |
| mlr5896 | 1,124243 |
| mll7010 | 1,1215815 |
| msl6121 | 1,119778 |
| msr3678 | 1,1171505 |
| mlr6559 | 1,111169 |
| mll6948 | 1,108547 |
| mlr1696 | 1,104472 |
| mlr9349 | 1,096278 |
| mll7088 | 1,096229 |
| mll1986 | 1,095431 |
| mll6391 | 1,094842 |
| msl8005 | 1,094135 |
| mll6249 | 1,087825 |
| mlr8224 | 1,08776 |
| mll6238 | 1,086703 |
| mlr5463 | 1,086256 |
| mll9310 | 1,085927 |
| mlr3905 | 1,085883 |
| mlr0414 | 1,0806605 |
| mll8115 | 1,077715 |
| mll4149 | 1,076478 |
| mll1914 | 1,073831 |
| msr2405 | 1,073649 |
| msl5783 | 1,07312 |
| mlr7355 | 1,070429 |
| msr5969 | 1,069716 |
| mlr9395 | 1,0657085 |
| mll2278 | 1,063926 |
| msr8514 | 1,062184 |
| mlr1904 | 1,058693 |
| mll3225 | 1,0567475 |
| mll4497 | 1,055066 |
| mll5840 | 1,053898 |
| mlr5216 | 1,052857 |
| mlr1800 | 1,049984 |
| mlr9207 | 1,04846 |
| mll0858 | 1,047545 |
| mlr8346 | 1,047446 |
| mlr6032 | 1,04684 |
| mlr6554 | 1,046724 |
| mlr2864 | 1,045711 |
| mlr9277 | 1,044028 |
| mlr0555 | 1,042825 |
| mlr6371 | 1,042421 |
| mlr8211 | 1,039605 |
| msr8681 | 1,039332 |
| mlr6768 | 1,038529 |
| mlr8141 | 1,038529 |
| mlr6163 | 1,035367 |
| mll1679 | 1,033799 |
| mlr6403 | 1,033361 |
| mll7035 | 1,033029 |
| mll5741 | 1,032318 |
| mlr3645 | 1,031442 |
| msl9071 | 1,03138 |
| mlr6656 | 1,031121 |
| mll4949 | 1,0301555 |
| mlr0229 | 1,025815 |
| mll1757 | 1,022392 |
| mlr6548 | 1,0202215 |
| msl1767 | 1,017371 |
| mlr3388 | 1,017312 |
| mlr0694 | 1,015756 |
| mll6882 | 1,0147 |
| mll9724 | 1,014675 |
| mlr6765 | 1,011663 |
| mlr8526 | 1,008381 |
| mlr2005 | 1,0042255 |
| msr4554 | 1,002655 |
| mlr6364 | 0,999659 |
| mlr1992 | 0,999036 |
| msl5982 | 0,995836 |
| mlr7051 | 0,992809 |
| mll8176 | 0,992002 |
| mlr9275 | 0,9890535 |
| msl6660 | 0,987029 |
| mlr7940 | 0,9868745 |
| mlr6551 | 0,984886 |
| mlr0825 | 0,983936 |
| mlr7217 | 0,978269 |
| mlr2718 | 0,977431 |
| mll3837 | 0,975779 |
| mll5614 | 0,974846 |
| mlr9539 | 0,974441 |
| mlr6156 | 0,973942 |
| mlr0532 | 0,972638 |
| mlr2358 | 0,972591 |
| mlr2034 | 0,972496 |
| mll5437 | 0,971873 |
| mll6926 | 0,970822 |
| mll1505 | 0,96908 |
| mlr9067 | 0,967825 |
| mlr7808 | 0,967515 |
| mlr0720 | 0,966042 |
| mll1872 | 0,964686 |
| mll9334 | 0,964262 |
| mll9177 | 0,962614 |
| mlr4633 | 0,961466 |
| mll7089 | 0,95818 |
| mlr2439 | 0,955977 |
| mll0450 | 0,9554335 |
| mlr7672 | 0,954694 |
| mlr8280 | 0,954205 |
| mll9677 | 0,953057 |
| mll7878 | 0,95106 |
| mll7312 | 0,950793 |
| mlr6674 | 0,94985 |
| mlr7847 | 0,9495975 |
| mll7303 | 0,947591 |
| mlr2711 | 0,9471 |
| mlr3455 | 0,9443195 |
| mll1137 | 0,944169 |
| mlr8018 | 0,944099 |
| msr8019 | 0,942 |
| mlr2406 | 0,941261 |
| mll5459 | 0,940648 |
| mll7203 | 0,937237 |
| mlr5923 | 0,936315 |
| mlr8533 | 0,9361455 |
| mlr3780 | 0,935301 |
| mlr6049 | 0,931968 |
| mll2445 | 0,931763 |
| msr1912 | 0,9308465 |
| mlr4314 | 0,929109 |
| mlr6095 | 0,927107 |
| mlr6758 | 0,926949 |
| mlr2316 | 0,924831 |
| mll7670 | 0,922151 |
| mlr3807 | 0,921657 |
| mlr7224 | 0,921128 |
| mlr3603 | 0,920219 |
| mll8137 | 0,9199565 |
| mll5758 | 0,91974 |
| mll7011 | 0,917253 |
| mll8078 | 0,916878 |
| mll5389 | 0,916779 |
| mll1791 | 0,916706 |
| mll7105 | 0,915097 |
| mlr2364 | 0,913754 |
| mll1962 | 0,913151 |
| mlr2365 | 0,912993 |
| mll5190 | 0,912872 |
| msr4734 | 0,912356 |
| mll6466 | 0,912239 |
| mll9132 | 0,91149 |
| mll3661 | 0,908513 |
| mll3094 | 0,9062275 |
| mll1903 | 0,9059 |
| mlr6557 | 0,9057395 |
| mll6040 | 0,905648 |
| mlr8522 | 0,904586 |
| mlr9540 | 0,901931 |
| mlr6756 | 0,901234 |
| mlr3740 | 0,900784 |
| mlr3359 | 0,89653 |
| mlr6241 | 0,89554 |
| mll9006 | 0,894658 |
| mll0645 | 0,894292 |
| mll5290 | 0,893527 |
| mll7200 | 0,893282 |
| mlr1984 | 0,888485 |
| mll3313 | 0,888181 |
| mll7617 | 0,8873455 |
| mll3453 | 0,887071 |
| mll0690 | 0,885804 |
| mlr1171 | 0,885448 |
| mll7072 | 0,88496 |
| msr8629 | 0,8812085 |
| mll3598 | 0,878094 |
| msr4045 | 0,878059 |
| mlr1907 | 0,875029 |
| mlr0145 | 0,872965 |
| mll5468 | 0,870832 |
| mlr0252 | 0,869257 |
| mll2355 | 0,86718 |
| mlr7740 | 0,866284 |
| mll9181 | 0,865875 |
| mlr0986 | 0,864731 |
| mll7443 | 0,862849 |
| mll4425 | 0,860783 |
| mll4659 | 0,859731 |
| mll7152 | 0,858177 |
| mlr1895 | 0,856125 |
| mll0115 | 0,852193 |
| mlr4464 | 0,851109 |
| mll7165 | 0,847831 |
| mll1779 | 0,847242 |
| mll3276 | 0,842423 |
| mll2425 | 0,842082 |
| mll5731 | 0,841936 |
| mlr9697 | 0,841741 |
| mlr4407 | 0,840643 |
| mll1617 | 0,840546 |
| mll0111 | 0,839837 |
| mll6782 | 0,837576 |
| mlr4804 | 0,836674 |
| mlr6969 | 0,836529 |
| mll1876 | 0,834147 |
| mlr1862 | 0,833032 |
| mlr0488 | 0,832009 |
| mll1037 | 0,830414 |
| mlr3784 | 0,828911 |
| mll4411 | 0,828647 |
| mlr4111 | 0,828105 |
| mlr4907 | 0,827902 |
| mlr5223 | 0,826676 |
| mll1960 | 0,826485 |
| mlr9675 | 0,823769 |
| mlr8341 | 0,823262 |
| msr9110 | 0,821135 |
| mll5958 | 0,820164 |
| mll0994 | 0,819816 |
| mlr4682 | 0,8191115 |
| msr9585 | 0,816904 |
| msl6009 | 0,815647 |
| mll3043 | 0,815212 |
| mlr7122 | 0,810646 |
| mlr3412 | 0,810376 |
| mll5688 | 0,809806 |
| mlr6792 | 0,808239 |
| mll9205 | 0,805977 |
| msl5920 | 0,803053 |
| mll4384 | 0,802721 |
| mll6619 | 0,800672 |
| mlr0478 | 0,800255 |
| mll7840 | 0,798288 |
| mll5960 | 0,797792 |
| msl8683 | 0,797704 |
| mll7978 | 0,794859 |
| mlr5977 | 0,793919 |
| mll6896 | 0,793728 |
| mll0381 | 0,793251 |
| mlr5662 | 0,79282 |
| msr9761 | 0,791722 |
| msr9570 | 0,790289 |
| msl9673 | 0,789974 |
| mlr6084 | 0,788646 |
| mlr9623 | 0,787581 |
| mll2525 | 0,7857315 |
| mll8313 | 0,782454 |
| mll3372 | 0,778463 |
| mlr4605 | 0,777426 |
| mll6887 | 0,776239 |
| mll4969 | 0,775067 |
| mlr9667 | 0,772048 |
| mlr6085 | 0,771422 |
| mlr3802 | 0,770875 |
| mll7330 | 0,7702105 |
| mll3481 | 0,765534 |
| mll7159 | 0,762986 |
| mlr1006 | 0,759915 |
| msl7783 | 0,75705 |
| mlr8014 | 0,756855 |
| mll1680 | 0,756839 |
| mlr6104 | 0,753264 |
| mlr5822 | 0,752974 |
| mlr7139 | 0,749685 |
| mll3044 | 0,749438 |
| mll7201 | 0,748593 |
| mlr1045 | 0,747136 |
| mlr9741 | 0,742378 |
| msr8578 | 0,741257 |
| mll3316 | 0,7384895 |
| mll9612 | 0,736164 |
| msl6052 | 0,733523 |
| mlr0493 | 0,7330595 |
| mlr6757 | 0,731717 |
| msl3084 | 0,730436 |
| msr5773 | 0,729492 |
| msl4838 | 0,728226 |
| mll1511 | 0,725142 |
| mlr6145 | 0,725108 |
| msr3806 | 0,7236455 |
| msr5979 | 0,72236 |
| mll6272 | 0,721373 |
| mlr3941 | 0,721241 |
| mlr6648 | 0,720362 |
| mlr0970 | 0,712346 |
| mll5829 | 0,704307 |
| mlr4086 | 0,7005 |
| mll4993 | 0,700231 |
| mlr6652 | 0,698143 |
| mll9114 | 0,693974 |
| msl8106 | 0,681842 |
| msr5722 | 0,6788 |
| mll8116 | 0,6753 |
| mll7417 | 0,660771 |
| msl4096 | 0,6587335 |
| mll7343 | 0,657147 |
| mlr4509 | 0,656463 |
| msl7040 | 0,648949 |
| mlr3516 | 0,646549 |
| mlr9279 | 0,646397 |
| msr1859 | 0,643907 |
| mlr2852 | 0,64305 |
| mll2335 | 0,635901 |
| msr3371 | 0,634814 |
| mll5431 | 0,634691 |
| msr5978 | 0,616751 |
| mlr6278 | 0,602375 |
| mlr8519 | 0,5656145 |
| msr6934 | 0,548896 |
| mlr2328 | 0,527004 |
| mlr9210 | 0,491743 |
| mlr2436 | 0,474077 |
| mll7187 | 0,445067 |
| mlr7003 | 0,357055 |
| mll9370 | 0,29639 |
| msr3770 | -0,6232695 |
| mll3384 | -0,661556 |
| mll5836 | -0,688816 |
| mll4125 | -0,691732 |
| mll7289 | -0,716362 |
| mll7812 | -0,728882 |
| mll4392 | -0,737663 |
| mlr9300 | -0,758514 |
| mlr0958 | -0,771554 |
| mlr5504 | -0,779526 |
| mlr0157 | -0,787003 |
| mll1296 | -0,788539 |
| mlr1968 | -0,792264 |
| mll1737 | -0,7936135 |
| mll3258 | -0,802198 |
| mlr3050 | -0,81427 |
| mlr1730 | -0,827556 |
| mlr2543 | -0,8280275 |
| mll7118 | -0,882485 |
| mll2655 | -0,885564 |
| mll2427 | -0,893843 |
| mll6776 | -0,893908 |
| mlr8325 | -0,913233 |
| mlr0211 | -0,916401 |
| mll4975 | -0,924333 |
| mll3259 | -0,926308 |
| msl5756 | -0,930066 |
| mlr0774 | -0,933631 |
| mll5195 | -0,946733 |
| mll3403 | -0,951072 |
| mll3903 | -0,975054 |
| mll7665 | -0,980171 |
| mll4732 | -0,988355 |
| mlr7915 | -0,991576 |
| mlr6292 | -0,992588 |
| mlr2792 | -0,993584 |
| mll1293 | -1,005988 |
| mlr0048 | -1,016107 |
| mll8252 | -1,02476 |
| mlr0100 | -1,024963 |
| mlr2557 | -1,037718 |
| mll2032 | -1,042333 |
| mll5393 | -1,045914 |
| mll0630 | -1,050929 |
| mll1289 | -1,052761 |
| mlr3212 | -1,05583 |
| mll6426 | -1,056373 |
| mll4333 | -1,058286 |
| mll3668 | -1,059669 |
| mll8295 | -1,0648075 |
| mlr7298 | -1,067163 |
| mll6464 | -1,070496 |
| mll6685 | -1,073282 |
| msr4212 | -1,073328 |
| mlr0783 | -1,079296 |
| mlr4864 | -1,082634 |
| msr6276 | -1,084991 |
| mlr1378 | -1,085144 |
| mlr6646 | -1,0946635 |
| mll0859 | -1,098725 |
| mlr5044 | -1,099461 |
| mlr7684 | -1,101842 |
| msl2014 | -1,107746 |
| mll4297 | -1,107991 |
| mlr8393 | -1,115326 |
| mlr2939 | -1,116615 |
| mll6519 | -1,121418 |
| msl5292 | -1,129591 |
| mll5651 | -1,133588 |
| mll7655 | -1,136716 |
| mll1351 | -1,138922 |
| mlr6488 | -1,140295 |
| mll7354 | -1,140475 |
| mlr3471 | -1,141651 |
| mlr2177 | -1,143878 |
| mlr3485 | -1,144638 |
| mll4265 | -1,1506185 |
| mll2492 | -1,156914 |
| mll6520 | -1,167976 |
| mll1719 | -1,169438 |
| mlr5631 | -1,169682 |
| mll0935 | -1,169895 |
| mll5565 | -1,176638 |
| mll5061 | -1,178824 |
| mlr4546 | -1,180294 |
| mlr4788 | -1,182065 |
| mlr8464 | -1,182409 |
| mlr5633 | -1,188316 |
| mll8488 | -1,189325 |
| mll0787 | -1,1893655 |
| mlr3465 | -1,193179 |
| mlr1512 | -1,195924 |
| mlr6494 | -1,195951 |
| mlr8273 | -1,195973 |
| mll5434 | -1,196259 |
| mll0800 | -1,198229 |
| mll1027 | -1,1990855 |
| mlr4763 | -1,200462 |
| mll0489 | -1,202658 |
| mlr6502 | -1,203997 |
| mll4976 | -1,209407 |
| mll1605 | -1,211085 |
| mlr3508 | -1,214456 |
| mlr3204 | -1,216126 |
| mlr5019 | -1,217673 |
| mlr4738 | -1,2179935 |
| mll8212 | -1,222815 |
| msl0722 | -1,223573 |
| mlr6670 | -1,228437 |
| mlr2198 | -1,228591 |
| mll0676 | -1,231072 |
| mlr0010 | -1,237603 |
| msl5725 | -1,238491 |
| mll2413 | -1,24515 |
| mlr8084 | -1,245244 |
| mll7934 | -1,247666 |
| msr5782 | -1,24881 |
| mll2564 | -1,250861 |
| mll7103 | -1,251813 |
| mlr7981 | -1,2590975 |
| mlr7030 | -1,261431 |
| mll5117 | -1,265875 |
| mlr0619 | -1,267319 |
| mll9653 | -1,26783 |
| msl6314 | -1,268181 |
| mlr7287 | -1,268563 |
| mll0900 | -1,268779 |
| mll1457 | -1,272053 |
| mll4450 | -1,272547 |
| mlr0543 | -1,27725 |
| mlr8270 | -1,278807 |
| mll5107 | -1,279148 |
| mlr7600 | -1,279737 |
| mll3040 | -1,285977 |
| mlr5413 | -1,291169 |
| mll4219 | -1,295166 |
| mlr0552 | -1,29517 |
| mlr2204 | -1,300881 |
| mlr0499 | -1,302534 |
| mlr3538 | -1,303837 |
| mll4550 | -1,304335 |
| mll3591 | -1,308401 |
| mlr1483 | -1,312351 |
| mll2519 | -1,320984 |
| mll5167 | -1,323408 |
| mll3385 | -1,325421 |
| mll9510 | -1,326302 |
| mlr6693 | -1,3314 |
| mlr3852 | -1,3328965 |
| mll4270 | -1,333132 |
| mlr9059 | -1,3374495 |
| mll5171 | -1,338076 |
| mlr1694 | -1,340032 |
| mll7648 | -1,342232 |
| msr6061 | -1,344028 |
| mll3738 | -1,345668 |
| mll0685 | -1,347393 |
| mlr2206 | -1,351765 |
| mll2867 | -1,352121 |
| mll3242 | -1,360806 |
| mlr7440 | -1,362055 |
| mlr5740 | -1,372404 |
| mll5659 | -1,372865 |
| mll5213 | -1,374391 |
| mll3287 | -1,3747425 |
| mll2368 | -1,3760815 |
| mlr7168 | -1,376504 |
| mll6996 | -1,378429 |
| mlr5505 | -1,378604 |
| mll3197 | -1,379984 |
| mll3998 | -1,380479 |
| mlr7616 | -1,381118 |
| mll2715 | -1,3814455 |
| mlr8405 | -1,384891 |
| mll0029 | -1,386073 |
| mlr5488 | -1,388287 |
| mlr5162 | -1,388443 |
| mll0416 | -1,393194 |
| mlr2201 | -1,398959 |
| mll6284 | -1,39954 |
| mlr5629 | -1,40731 |
| mll8482 | -1,408872 |
| msr8102 | -1,410777 |
| mlr5751 | -1,412734 |
| mlr7475 | -1,417076 |
| msl2982 | -1,417542 |
| mll5423 | -1,4200465 |
| mlr4884 | -1,42234 |
| mlr2935 | -1,425484 |
| mll5502 | -1,426011 |
| mlr5013 | -1,426391 |
| mll3163 | -1,429291 |
| mll1765 | -1,42938 |
| msr7277 | -1,430107 |
| mll3901 | -1,430123 |
| mll8447 | -1,430987 |
| mll5191 | -1,431025 |
| msl1949 | -1,434475 |
| msl4031 | -1,437695 |
| mll4892 | -1,4383965 |
| mll0934 | -1,439713 |
| msl0261 | -1,442756 |
| msl6332 | -1,4436575 |
| mlr2897 | -1,44548 |
| mlr3533 | -1,448463 |
| msl0055 | -1,450383 |
| mlr7267 | -1,451774 |
| mlr5400 | -1,454385 |
| mll4470 | -1,456813 |
| mll1848 | -1,457452 |
| mll5286 | -1,461171 |
| mll8348 | -1,461221 |
| mlr5208 | -1,46254 |
| mlr7916 | -1,462992 |
| mlr4941 | -1,465688 |
| mlr5498 | -1,466409 |
| mlr5678 | -1,4664885 |
| mlr7453 | -1,466626 |
| mlr8162 | -1,467002 |
| mlr7802 | -1,467945 |
| mll4390 | -1,469547 |
| mlr2965 | -1,469596 |
| mll0788 | -1,469664 |
| mlr3300 | -1,470116 |
| mlr2027 | -1,475177 |
| mll2586 | -1,47559 |
| mll8172 | -1,476116 |
| mlr0908 | -1,476241 |
| mlr5554 | -1,476607 |
| mll3737 | -1,476665 |
| mlr2763 | -1,47881 |
| mlr8551 | -1,479023 |
| mll8388 | -1,480754 |
| mlr3463 | -1,480834 |
| mll5577 | -1,485249 |
| mll8244 | -1,485774 |
| mlr7652 | -1,487318 |
| mll6251 | -1,488173 |
| mlr0906 | -1,489231 |
| msl4978 | -1,4904325 |
| mlr4670 | -1,491064 |
| mlr9157 | -1,494618 |
| mll3324 | -1,496792 |
| mlr0396 | -1,5002115 |
| mlr4849 | -1,500687 |
| mll0730 | -1,502196 |
| mlr5610 | -1,502743 |
| mll0869 | -1,503509 |
| mlr5988 | -1,50388 |
| mlr3583 | -1,509424 |
| mlr5697 | -1,510906 |
| mll8434 | -1,512983 |
| mlr7483 | -1,513443 |
| mll2475 | -1,513628 |
| mll3791 | -1,5141635 |
| mll4312 | -1,514293 |
| mll0206 | -1,5143135 |
| mlr0125 | -1,5153615 |
| mlr0812 | -1,517742 |
| mll3228 | -1,520189 |
| mll4363 | -1,520414 |
| mlr7806 | -1,525905 |
| mll1350 | -1,534177 |
| msr9113 | -1,53506 |
| mll5493 | -1,535081 |
| mlr9681 | -1,536427 |
| mlr7452 | -1,536623 |
| mlr3684 | -1,538371 |
| mlr7099 | -1,541133 |
| mlr0549 | -1,541714 |
| mlr7556 | -1,543254 |
| mlr5698 | -1,543859 |
| mll1399 | -1,544102 |
| mll1362 | -1,545362 |
| mll1627 | -1,546348 |
| mll4250 | -1,548106 |
| mll3023 | -1,548621 |
| mll6253 | -1,550899 |
| mlr0033 | -1,55297 |
| mlr3785 | -1,5534645 |
| mlr2630 | -1,5543275 |
| mlr4770 | -1,554977 |
| mll8451 | -1,556478 |
| msl0491 | -1,557043 |
| msl2011 | -1,557691 |
| msl8646 | -1,560779 |
| mll1538 | -1,562263 |
| mll5964 | -1,5630455 |
| mll4618 | -1,563675 |
| mlr4932 | -1,5670615 |
| mll2850 | -1,5678335 |
| mlr3524 | -1,568541 |
| mll2537 | -1,568976 |
| mlr5036 | -1,569073 |
| mlr0925 | -1,571628 |
| mlr7504 | -1,574211 |
| mlr2893 | -1,578356 |
| mll3746 | -1,579809 |
| mlr4394 | -1,581671 |
| mlr3749 | -1,5819265 |
| mll3559 | -1,591848 |
| mll1950 | -1,592673 |
| mll2869 | -1,596539 |
| msr4725 | -1,598937 |
| mlr0874 | -1,599436 |
| mlr7777 | -1,602467 |
| msl0779 | -1,603437 |
| mlr3301 | -1,605967 |
| mlr7769 | -1,607434 |
| mll8158 | -1,608739 |
| mlr8407 | -1,608943 |
| mll5123 | -1,60969 |
| mlr3913 | -1,611475 |
| mlr5597 | -1,612347 |
| mll0051 | -1,6127785 |
| msr5728 | -1,61428 |
| mlr7582 | -1,615999 |
| mll0208 | -1,618067 |
| mll4008 | -1,621213 |
| mlr2480 | -1,621581 |
| mlr4626 | -1,622906 |
| mlr2732 | -1,625408 |
| mll0711 | -1,62659 |
| mlr0329 | -1,627197 |
| mll3859 | -1,627511 |
| mlr5543 | -1,627584 |
| mll4442 | -1,6278885 |
| mlr3811 | -1,628678 |
| mlr5420 | -1,629178 |
| mlr3056 | -1,631631 |
| mll6255 | -1,6348005 |
| mlr3880 | -1,636155 |
| mlr1634 | -1,637024 |
| mlr1282 | -1,6421555 |
| mll2036 | -1,642456 |
| msl4631 | -1,646419 |
| mlr4745 | -1,64798 |
| mll6897 | -1,648714 |
| mlr4237 | -1,650046 |
| mll4306 | -1,650531 |
| mll4019 | -1,653483 |
| mlr2795 | -1,654005 |
| mlr5275 | -1,656188 |
| mll6243 | -1,6584585 |
| mll6499 | -1,658638 |
| mlr5175 | -1,6589245 |
| mlr4643 | -1,661327 |
| mll5415 | -1,665539 |
| mlr1039 | -1,667297 |
| mlr0698 | -1,6720855 |
| mll1346 | -1,673438 |
| mlr8360 | -1,674932 |
| mll0861 | -1,676923 |
| mlr0331 | -1,677792 |
| mlr1387 | -1,678726 |
| mlr0912 | -1,679204 |
| mll0618 | -1,6812325 |
| mlr6990 | -1,685717 |
| mlr4335 | -1,688041 |
| mlr3548 | -1,688223 |
| mlr5260 | -1,688851 |
| mlr1120 | -1,690082 |
| mll0106 | -1,6907435 |
| mll1167 | -1,697404 |
| mll7454 | -1,698414 |
| mlr7339 | -1,6987345 |
| mll4858 | -1,702272 |
| msl3868 | -1,703486 |
| mll4009 | -1,704948 |
| mlr1383 | -1,705046 |
| msl4750 | -1,7080765 |
| mll0653 | -1,709538 |
| mll3551 | -1,711948 |
| mll1417 | -1,713341 |
| mll4931 | -1,714455 |
| mll0579 | -1,714531 |
| mll9009 | -1,715845 |
| mll6677 | -1,716497 |
| mlr4491 | -1,717907 |
| mll1228 | -1,718287 |
| mll4923 | -1,718787 |
| mll3564 | -1,719785 |
| mlr7266 | -1,721027 |
| mll5398 | -1,722519 |
| mlr7500 | -1,72333 |
| mll5620 | -1,724399 |
| mll3039 | -1,724835 |
| mll1880 | -1,728191 |
| mll0362 | -1,728398 |
| mll4176 | -1,731086 |
| mlr7560 | -1,731742 |
| mll0888 | -1,731761 |
| mlr0615 | -1,732656 |
| msl6451 | -1,734591 |
| mll2989 | -1,736433 |
| mll3743 | -1,737 |
| msl5425 | -1,738635 |
| mll5321 | -1,741303 |
| mll8258 | -1,741681 |
| mlr1231 | -1,7436525 |
| mll3362 | -1,744273 |
| msl5079 | -1,7463105 |
| mll6504 | -1,747043 |
| mll8131 | -1,748347 |
| mlr6692 | -1,751435 |
| mll0899 | -1,751729 |
| mlr4100 | -1,752062 |
| mlr0374 | -1,752114 |
| mll1920 | -1,752856 |
| msl1345 | -1,753309 |
| mll1093 | -1,758262 |
| mlr4646 | -1,758926 |
| mlr2813 | -1,759325 |
| mlr3522 | -1,760145 |
| mll5573 | -1,760961 |
| mll0951 | -1,7615045 |
| mlr0011 | -1,763075 |
| mll4989 | -1,76553 |
| mlr5480 | -1,766926 |
| mlr0385 | -1,771229 |
| mlr0808 | -1,776763 |
| mlr1298 | -1,7778045 |
| mlr1614 | -1,779342 |
| mlr0614 | -1,781804 |
| mlr0305 | -1,782024 |
| mll5634 | -1,784899 |
| mll3824 | -1,785623 |
| mll2069 | -1,788975 |
| mlr4672 | -1,7890085 |
| mlr3753 | -1,789723 |
| mll1587 | -1,789917 |
| mll1589 | -1,79415 |
| mll3108 | -1,794713 |
| mll3656 | -1,795351 |
| mll1553 | -1,795564 |
| mlr5989 | -1,795666 |
| mlr3933 | -1,795735 |
| msr1301 | -1,796058 |
| mlr5262 | -1,798234 |
| mlr7270 | -1,800545 |
| mlr8052 | -1,803375 |
| mll4332 | -1,804912 |
| mlr3257 | -1,805545 |
| mll2628 | -1,805624 |
| mll5052 | -1,806127 |
| mll2260 | -1,807512 |
| mll0526 | -1,808867 |
| msr5817 | -1,809666 |
| mlr5747 | -1,813207 |
| mlr5643 | -1,813647 |
| mll4063 | -1,814394 |
| mll0832 | -1,815115 |
| mll4959 | -1,8198 |
| msr7914 | -1,821037 |
| mlr8401 | -1,821484 |
| mlr3252 | -1,821936 |
| mll0065 | -1,823372 |
| mll4453 | -1,825314 |
| mlr7821 | -1,826402 |
| mll0141 | -1,826557 |
| msl0567 | -1,8277805 |
| mll5998 | -1,828667 |
| mll1455 | -1,828696 |
| mlr8392 | -1,8351375 |
| mll7547 | -1,837967 |
| mlr6852 | -1,838645 |
| mlr7649 | -1,839015 |
| mlr6658 | -1,839371 |
| mll2847 | -1,841858 |
| mll5681 | -1,842793 |
| mll0343 | -1,842847 |
| mlr6817 | -1,843094 |
| msl6380 | -1,844764 |
| mll7865 | -1,846537 |
| mll3934 | -1,850101 |
| mlr2023 | -1,850367 |
| mll4416 | -1,8505215 |
| mll5047 | -1,852566 |
| mlr7857 | -1,857391 |
| msr1502 | -1,857878 |
| mll5499 | -1,85788 |
| mlr2793 | -1,861477 |
| mlr5533 | -1,863683 |
| mlr6691 | -1,871384 |
| mlr1381 | -1,875453 |
| mll3104 | -1,875982 |
| mll5323 | -1,877901 |
| mll7959 | -1,880692 |
| mll1318 | -1,88129 |
| mll4859 | -1,882116 |
| mll5110 | -1,886025 |
| mll1459 | -1,886472 |
| msl1536 | -1,888212 |
| mlr7588 | -1,889107 |
| mlr0554 | -1,89057 |
| mlr1020 | -1,892449 |
| mlr7768 | -1,892826 |
| mlr8284 | -1,895246 |
| mlr1579 | -1,8956205 |
| mll3873 | -1,89898 |
| mll1660 | -1,900886 |
| mlr2774 | -1,902509 |
| mll3573 | -1,9050175 |
| mlr2085 | -1,905078 |
| mlr9258 | -1,90606 |
| mlr7635 | -1,906217 |
| mll1341 | -1,906544 |
| mlr7697 | -1,907421 |
| mlr3855 | -1,909224 |
| mll3821 | -1,909671 |
| mlr5064 | -1,9106605 |
| mlr1304 | -1,913277 |
| mlr6574 | -1,914995 |
| mll2478 | -1,915847 |
| mll2602 | -1,916778 |
| mlr0905 | -1,916946 |
| mlr4290 | -1,917122 |
| mlr4536 | -1,917282 |
| mll3560 | -1,91768 |
| mlr2909 | -1,918647 |
| msl7335 | -1,9208325 |
| mll1048 | -1,925197 |
| mll2664 | -1,925907 |
| mlr0096 | -1,926977 |
| mlr4960 | -1,927647 |
| mll5233 | -1,928144 |
| mlr2667 | -1,929665 |
| mll7843 | -1,9296925 |
| mlr3482 | -1,932979 |
| mlr4744 | -1,933094 |
| mll7729 | -1,933424 |
| mll6383 | -1,934761 |
| mlr1388 | -1,9355 |
| mll9348 | -1,935613 |
| mlr2444 | -1,9364995 |
| mlr2805 | -1,939461 |
| mll4444 | -1,946378 |
| mll0715 | -1,94807 |
| mll4301 | -1,949549 |
| mll1835 | -1,949608 |
| mll1080 | -1,950404 |
| mlr0039 | -1,950751 |
| msr4573 | -1,951473 |
| msl3747 | -1,954813 |
| mll4305 | -1,955544 |
| mlr5628 | -1,955719 |
| mll1621 | -1,957626 |
| mll6066 | -1,959302 |
| mlr7856 | -1,961807 |
| mlr3847 | -1,9627935 |
| mll0405 | -1,969971 |
| mlr7272 | -1,971978 |
| mlr2581 | -1,9723935 |
| mll6498 | -1,974429 |
| mll4479 | -1,974864 |
| mll0610 | -1,975773 |
| mll2703 | -1,975825 |
| mll1611 | -1,977584 |
| mlr8228 | -1,978972 |
| mlr0872 | -1,979258 |
| mlr2620 | -1,979719 |
| mll5422 | -1,98053 |
| mlr7404 | -1,980605 |
| mlr7778 | -1,980919 |
| msr3483 | -1,981417 |
| mll2080 | -1,983012 |
| mlr7933 | -1,9847 |
| mll3970 | -1,9856925 |
| mll0710 | -1,986643 |
| mll3168 | -1,986674 |
| mlr1664 | -1,987484 |
| mll1067 | -1,988236 |
| mlr4812 | -1,989996 |
| mlr5010 | -1,990318 |
| mlr8153 | -1,990402 |
| mlr9176 | -1,9920875 |
| mlr7686 | -1,993878 |
| mll1602 | -1,995262 |
| mlr5557 | -1,9963405 |
| mlr3497 | -1,997206 |
| mll1724 | -1,997452 |
| mll6033 | -1,998758 |
| mll0028 | -1,999749 |
| msr4424 | -2,001993 |
| mlr7748 | -2,002214 |
| mlr8465 | -2,004379 |
| mll5569 | -2,005 |
| mlr7426 | -2,006724 |
| mll1292 | -2,009037 |
| mll3563 | -2,011914 |
| mll9244 | -2,012136 |
| mlr8352 | -2,013844 |
| mlr9242 | -2,014378 |
| mll6611 | -2,017071 |
| mlr2516 | -2,018327 |
| mlr0937 | -2,020395 |
| mlr4951 | -2,021622 |
| mll7528 | -2,022542 |
| mlr3332 | -2,0225525 |
| mll1145 | -2,023358 |
| mll0501 | -2,025324 |
| mll3877 | -2,02575 |
| mll1644 | -2,026567 |
| mll4272 | -2,027329 |
| mlr9253 | -2,028106 |
| mlr3285 | -2,028282 |
| mll4060 | -2,029391 |
| mll0792 | -2,030968 |
| mlr0629 | -2,031588 |
| mlr8409 | -2,0347575 |
| mlr5534 | -2,035508 |
| mlr0213 | -2,038659 |
| mlr2095 | -2,040354 |
| mll3380 | -2,040434 |
| mll6355 | -2,0405345 |
| mlr0219 | -2,041159 |
| mlr2696 | -2,041735 |
| mll2881 | -2,042437 |
| mlr1131 | -2,045226 |
| mlr5515 | -2,047363 |
| mll3025 | -2,053468 |
| mlr2747 | -2,056768 |
| mll4471 | -2,060318 |
| mll9104 | -2,062014 |
| mlr7688 | -2,0621265 |
| mlr6684 | -2,063345 |
| mlr7900 | -2,0638415 |
| mlr6539 | -2,067366 |
| msl5749 | -2,067834 |
| mll3581 | -2,069838 |
| mlr8225 | -2,070819 |
| mll4065 | -2,0760325 |
| mlr4370 | -2,077043 |
| mlr7849 | -2,078002 |
| mll3090 | -2,0807955 |
| mlr6681 | -2,081069 |
| mlr1178 | -2,081097 |
| mlr2942 | -2,08281 |
| mlr5623 | -2,083638 |
| mlr8466 | -2,084113 |
| mlr4368 | -2,087095 |
| mll0833 | -2,087704 |
| mll1519 | -2,090713 |
| mlr8385 | -2,090724 |
| mlr0073 | -2,0928205 |
| mll4299 | -2,09579 |
| mlr7920 | -2,0962925 |
| mlr2565 | -2,097255 |
| mll1256 | -2,0973235 |
| mlr2024 | -2,098615 |
| mll4886 | -2,098838 |
| mll4906 | -2,099876 |
| mll1451 | -2,101295 |
| mlr7771 | -2,1021055 |
| mll8575 | -2,102821 |
| mlr3584 | -2,103368 |
| mlr3786 | -2,103762 |
| mlr0551 | -2,104288 |
| mll0502 | -2,104842 |
| mll3532 | -2,105213 |
| mlr7447 | -2,105336 |
| mll9087 | -2,106192 |
| mll4752 | -2,10726 |
| mll4634 | -2,108571 |
| mlr4882 | -2,109319 |
| mlr4647 | -2,112762 |
| mlr5381 | -2,113412 |
| mlr0384 | -2,116599 |
| mll2287 | -2,116772 |
| mlr4776 | -2,117428 |
| mlr4739 | -2,118492 |
| msl0655 | -2,122216 |
| mll2568 | -2,122366 |
| mll1125 | -2,122591 |
| mll6291 | -2,124228 |
| mlr0349 | -2,126159 |
| mlr5016 | -2,128532 |
| mlr7256 | -2,129255 |
| mll1184 | -2,130819 |
| mlr0118 | -2,131669 |
| mlr7622 | -2,131976 |
| mlr1379 | -2,13225 |
| mlr5268 | -2,134158 |
| mlr6929 | -2,135805 |
| mll2888 | -2,137855 |
| mlr4795 | -2,139416 |
| mlr0199 | -2,143081 |
| mlr4841 | -2,144975 |
| mll1656 | -2,145711 |
| mll6338 | -2,14818 |
| mll6970 | -2,149257 |
| mlr1073 | -2,151214 |
| mlr4078 | -2,151905 |
| mlr7477 | -2,155817 |
| msr3768 | -2,156163 |
| mlr4380 | -2,157014 |
| mlr1135 | -2,159 |
| msl9291 | -2,15961 |
| mlr3809 | -2,162226 |
| mll0713 | -2,164012 |
| mlr1179 | -2,164701 |
| mll5576 | -2,167215 |
| mll1183 | -2,167544 |
| mlr6727 | -2,1692955 |
| mll0183 | -2,1715505 |
| mll0801 | -2,17276 |
| mlr9260 | -2,174691 |
| mll3361 | -2,175816 |
| mlr8032 | -2,177484 |
| mlr0324 | -2,177547 |
| mlr4072 | -2,179232 |
| msl0793 | -2,1798915 |
| mlr3014 | -2,180556 |
| mlr0739 | -2,18205 |
| mlr0883 | -2,184007 |
| mll5269 | -2,185488 |
| mll7536 | -2,185928 |
| mll4199 | -2,190037 |
| mlr5364 | -2,190422 |
| mll4523 | -2,193032 |
| mlr4350 | -2,195124 |
| mlr5419 | -2,196789 |
| mlr8250 | -2,197168 |
| mlr6427 | -2,197216 |
| mlr0210 | -2,199025 |
| mll4934 | -2,201684 |
| mlr6925 | -2,202295 |
| mll9560 | -2,203544 |
| mll1454 | -2,204001 |
| mlr3097 | -2,207794 |
| mlr6159 | -2,208015 |
| mll8456 | -2,209787 |
| mll6378 | -2,210228 |
| mll4059 | -2,21051 |
| mll7734 | -2,211946 |
| mlr6774 | -2,212217 |
| mlr6946 | -2,213216 |
| mll7642 | -2,2151265 |
| mll1552 | -2,216839 |
| mlr8242 | -2,217918 |
| msl4038 | -2,218905 |
| mll3001 | -2,219794 |
| mll6688 | -2,221009 |
| mlr7868 | -2,2211755 |
| mll5001 | -2,221768 |
| mlr5559 | -2,2238985 |
| mlr5527 | -2,226674 |
| mll6923 | -2,226951 |
| mll4372 | -2,227207 |
| mll1545 | -2,229042 |
| mll2884 | -2,229159 |
| mlr2502 | -2,229189 |
| mll4924 | -2,230937 |
| mlr9522 | -2,232767 |
| mll8334 | -2,232895 |
| mll1629 | -2,2338 |
| mll7348 | -2,2339 |
| msr4423 | -2,236285 |
| mlr9649 | -2,240058 |
| mll7580 | -2,2412795 |
| mll6718 | -2,241501 |
| mlr2924 | -2,241942 |
| mlr7799 | -2,2433 |
| mll2846 | -2,2437085 |
| msr0098 | -2,244036 |
| mll3879 | -2,244751 |
| mlr3114 | -2,244997 |
| mll0916 | -2,245539 |
| mlr3262 | -2,245699 |
| mll8278 | -2,2465 |
| mlr6203 | -2,249057 |
| mlr6633 | -2,24986 |
| mll4273 | -2,251175 |
| mlr1098 | -2,251487 |
| mll6433 | -2,252805 |
| mll4070 | -2,252971 |
| mlr0170 | -2,253414 |
| mll4454 | -2,254797 |
| mlr3256 | -2,2582015 |
| mlr8427 | -2,258948 |
| mll4889 | -2,261473 |
| mll4751 | -2,266741 |
| mll0767 | -2,2668565 |
| mlr7155 | -2,267276 |
| mlr6429 | -2,267901 |
| mll0566 | -2,268057 |
| mlr2938 | -2,269899 |
| mlr0286 | -2,271564 |
| mll4753 | -2,271648 |
| mlr4639 | -2,272682 |
| mll3890 | -2,273417 |
| mll4040 | -2,273979 |
| mlr4671 | -2,275263 |
| mlr2680 | -2,276624 |
| mll5656 | -2,276749 |
| mll7581 | -2,277153 |
| mll3460 | -2,277517 |
| msr0960 | -2,27829 |
| mlr3853 | -2,284194 |
| mll3976 | -2,285918 |
| mlr2987 | -2,28632 |
| mlr2775 | -2,288498 |
| mlr0322 | -2,289562 |
| mll5341 | -2,290052 |
| mll1643 | -2,2910915 |
| mlr6947 | -2,293101 |
| mlr6117 | -2,294755 |
| mll0823 | -2,295256 |
| mll3884 | -2,298813 |
| mll1361 | -2,299048 |
| mll4048 | -2,2994 |
| mll4302 | -2,299793 |
| mlr2298 | -2,304031 |
| mlr4269 | -2,304238 |
| mll5564 | -2,305213 |
| mlr7259 | -2,3065865 |
| msr0293 | -2,306892 |
| mll7421 | -2,308348 |
| mll2673 | -2,313882 |
| mll5204 | -2,313933 |
| mlr7913 | -2,314471 |
| mlr5018 | -2,314826 |
| mll3065 | -2,315043 |
| mll0871 | -2,315216 |
| mll7730 | -2,315817 |
| mll4781 | -2,319448 |
| mlr5639 | -2,320054 |
| mlr5133 | -2,3219665 |
| mll3792 | -2,32261 |
| mll4293 | -2,324816 |
| mll4271 | -2,326527 |
| mlr7813 | -2,327259 |
| mlr3264 | -2,329592 |
| mlr0400 | -2,329797 |
| mlr0562 | -2,333799 |
| mlr3027 | -2,334921 |
| mlr9267 | -2,33593 |
| mlr4381 | -2,337906 |
| mll5780 | -2,339076 |
| mll5088 | -2,339677 |
| mlr4846 | -2,3452805 |
| mlr5014 | -2,346664 |
| mll3722 | -2,347254 |
| mlr2771 | -2,34777 |
| mll3781 | -2,349556 |
| mlr0401 | -2,350719 |
| mll1812 | -2,352963 |
| mlr4157 | -2,354769 |
| mlr4721 | -2,354857 |
| mlr3157 | -2,357133 |
| mlr7559 | -2,359332 |
| mlr1177 | -2,360013 |
| mlr0190 | -2,363901 |
| mll3505 | -2,364039 |
| mll7573 | -2,3642905 |
| msr0370 | -2,3684425 |
| mll6479 | -2,36863 |
| mlr0283 | -2,368732 |
| mll3872 | -2,368761 |
| mlr3649 | -2,369111 |
| mlr5626 | -2,369197 |
| mll5809 | -2,369757 |
| mll4807 | -2,370134 |
| mll9517 | -2,371107 |
| mlr0662 | -2,371252 |
| mll5285 | -2,371526 |
| mll4918 | -2,373273 |
| mlr1186 | -2,373582 |
| mll5212 | -2,374217 |
| mlr7553 | -2,376777 |
| mlr5008 | -2,379109 |
| mlr3848 | -2,379654 |
| msl8272 | -2,380739 |
| mlr1729 | -2,388715 |
| msr0304 | -2,389156 |
| mlr0399 | -2,389242 |
| mll1090 | -2,392283 |
| mll8511 | -2,393723 |
| mll7488 | -2,394592 |
| mlr7597 | -2,394733 |
| mlr7906 | -2,394845 |
| mlr3019 | -2,40151 |
| mll3944 | -2,404286 |
| mll4330 | -2,4052085 |
| mlr7505 | -2,40649 |
| mlr3487 | -2,408835 |
| mlr5273 | -2,409928 |
| mlr3210 | -2,41074 |
| mlr3155 | -2,411434 |
| mll1028 | -2,412692 |
| mlr4761 | -2,414185 |
| mlr5738 | -2,419354 |
| mll1421 | -2,419513 |
| mll3536 | -2,421888 |
| mlr6928 | -2,425586 |
| mll8486 | -2,425811 |
| mll6829 | -2,42655 |
| mlr0901 | -2,42772 |
| mlr5404 | -2,427827 |
| mlr6409 | -2,428061 |
| mlr0519 | -2,432486 |
| mlr7589 | -2,432525 |
| mll1921 | -2,432794 |
| mlr7527 | -2,435059 |
| mll5649 | -2,436518 |
| mlr9246 | -2,436609 |
| msl9578 | -2,438521 |
| mll3195 | -2,439384 |
| mlr7455 | -2,439805 |
| mll0063 | -2,443417 |
| mll0074 | -2,4436975 |
| mlr9252 | -2,445448 |
| mll0724 | -2,447564 |
| mlr2471 | -2,4493905 |
| mlr6601 | -2,451344 |
| mll6921 | -2,452326 |
| mlr5553 | -2,453762 |
| mlr0333 | -2,454195 |
| mll4217 | -2,454243 |
| mll3321 | -2,458469 |
| mll9315 | -2,458917 |
| mll1365 | -2,459612 |
| mll2895 | -2,462757 |
| mll2499 | -2,464571 |
| mlr4209 | -2,467023 |
| mlr5253 | -2,468675 |
| msl9074 | -2,471714 |
| mll4433 | -2,472932 |
| mlr1535 | -2,4731 |
| mlr1375 | -2,473359 |
| mll4266 | -2,475696 |
| mlr1236 | -2,477748 |
| mlr2910 | -2,481092 |
| mlr0284 | -2,481518 |
| mll8460 | -2,481678 |
| mll3196 | -2,483694 |
| mll0609 | -2,48473 |
| mlr4452 | -2,486639 |
| mll2809 | -2,486962 |
| mlr5267 | -2,487031 |
| mlr0169 | -2,489087 |
| mll2073 | -2,48914 |
| mlr0660 | -2,490246 |
| mlr7549 | -2,490383 |
| mlr2782 | -2,492783 |
| mlr8275 | -2,49279 |
| mlr5249 | -2,495055 |
| mll7861 | -2,49818 |
| mlr9256 | -2,4985015 |
| mll6632 | -2,499261 |
| mlr3926 | -2,50387 |
| mlr2740 | -2,505341 |
| mlr3754 | -2,508616 |
| mll4408 | -2,508777 |
| mll3952 | -2,509309 |
| mll1584 | -2,510856 |
| mlr7585 | -2,51205 |
| mll3891 | -2,513367 |
| mlr4760 | -2,513442 |
| mll0155 | -2,5142655 |
| mlr5164 | -2,515003 |
| mlr0187 | -2,515361 |
| mll4832 | -2,519779 |
| mll2226 | -2,522648 |
| mll4109 | -2,522726 |
| mll7290 | -2,523512 |
| mll6957 | -2,523543 |
| mlr5276 | -2,523636 |
| mll6459 | -2,523846 |
| mll7653 | -2,525136 |
| mlr7805 | -2,5276455 |
| mlr2633 | -2,531925 |
| mll6606 | -2,532011 |
| mlr0180 | -2,5325315 |
| mll8354 | -2,533448 |
| mll4534 | -2,534531 |
| mll3592 | -2,534653 |
| mll1934 | -2,535673 |
| mlr9249 | -2,535686 |
| mlr5181 | -2,535941 |
| mlr0864 | -2,5382645 |
| mll4472 | -2,53883 |
| mll0907 | -2,539411 |
| mlr2761 | -2,540301 |
| mlr5517 | -2,543701 |
| mlr3216 | -2,544471 |
| mlr5630 | -2,545367 |
| mll3562 | -2,54888 |
| mll1070 | -2,551246 |
| mlr7503 | -2,552199 |
| mll5335 | -2,553581 |
| mll7514 | -2,554996 |
| msl4649 | -2,555041 |
| mll0633 | -2,557631 |
| mlr5523 | -2,559362 |
| mll3580 | -2,55942 |
| mlr8474 | -2,563134 |
| mlr1377 | -2,563555 |
| mlr0514 | -2,564487 |
| mll1625 | -2,566231 |
| mlr7756 | -2,56727 |
| mll5202 | -2,5682015 |
| mll4068 | -2,5708775 |
| mlr0102 | -2,572161 |
| mlr5015 | -2,572416 |
| mlr0376 | -2,57298 |
| mll5193 | -2,577947 |
| mlr7570 | -2,578377 |
| mll2297 | -2,578426 |
| mlr4292 | -2,582277 |
| mlr9108 | -2,582297 |
| mll8435 | -2,584348 |
| mlr6716 | -2,584793 |
| mll3461 | -2,585239 |
| mlr0032 | -2,589244 |
| mll2060 | -2,594916 |
| mlr8191 | -2,596942 |
| mlr5654 | -2,597851 |
| mlr1174 | -2,601012 |
| mll4263 | -2,60387 |
| mll1951 | -2,6068895 |
| mlr0398 | -2,6097435 |
| mll4933 | -2,610577 |
| mll7731 | -2,615371 |
| mlr5642 | -2,615407 |
| mll0818 | -2,617479 |
| mll4473 | -2,620541 |
| mlr1123 | -2,621985 |
| mll7961 | -2,622776 |
| mlr2682 | -2,62285 |
| mll4485 | -2,622867 |
| mll0679 | -2,622968 |
| mll1902 | -2,623848 |
| mll4077 | -2,625954 |
| mll7640 | -2,626616 |
| mlr9236 | -2,627951 |
| mlr2941 | -2,629874 |
| mll8240 | -2,6299105 |
| mll5317 | -2,631435 |
| mlr5274 | -2,635271 |
| mll9542 | -2,635639 |
| mlr8412 | -2,637238 |
| msr1055 | -2,639961 |
| mlr3148 | -2,6400175 |
| mlr5514 | -2,640644 |
| mlr8492 | -2,640649 |
| mlr4239 | -2,643486 |
| mll4486 | -2,645572 |
| msl0067 | -2,647054 |
| mlr4741 | -2,653098 |
| mll4283 | -2,655416 |
| mlr3495 | -2,655573 |
| mll4112 | -2,6562685 |
| mll4443 | -2,657557 |
| mll4310 | -2,65825 |
| mll4298 | -2,6585435 |
| mlr7584 | -2,659411 |
| mlr6118 | -2,663508 |
| mlr6204 | -2,664737 |
| msr3579 | -2,665506 |
| mll1102 | -2,667978 |
| mll4888 | -2,668491 |
| mll1278 | -2,6691725 |
| mll1661 | -2,670139 |
| mlr5547 | -2,6703645 |
| mll3198 | -2,671297 |
| mlr6158 | -2,672688 |
| mlr3554 | -2,6728975 |
| mlr5526 | -2,673687 |
| mll1069 | -2,675188 |
| mll4920 | -2,67613 |
| mll0728 | -2,677181 |
| mll0224 | -2,677202 |
| mlr7876 | -2,67827 |
| mlr5652 | -2,680677 |
| mll1727 | -2,681608 |
| mlr4742 | -2,683251 |
| mlr7854 | -2,684262 |
| mlr8269 | -2,684858 |
| mll3812 | -2,685203 |
| msl4039 | -2,6916165 |
| msl0677 | -2,692954 |
| mlr3166 | -2,694105 |
| mlr1112 | -2,696206 |
| mlr1581 | -2,6968025 |
| mll7835 | -2,6969985 |
| mlr5184 | -2,697033 |
| mll1087 | -2,697173 |
| mlr7551 | -2,697201 |
| mll9167 | -2,6972365 |
| mlr5144 | -2,697452 |
| msr0303 | -2,698322 |
| mlr0009 | -2,7002345 |
| msr2658 | -2,702452 |
| mlr9251 | -2,703612 |
| mlr5536 | -2,705391 |
| mll6353 | -2,711379 |
| mlr4810 | -2,711844 |
| msr6108 | -2,712739 |
| mll2877 | -2,714767 |
| mll2885 | -2,715508 |
| mlr3423 | -2,718056 |
| mll4842 | -2,718246 |
| msr2684 | -2,718441 |
| mlr0613 | -2,718852 |
| mll9093 | -2,719116 |
| mlr7403 | -2,721387 |
| mll4259 | -2,721482 |
| mll5657 | -2,7229 |
| mll6256 | -2,723061 |
| mll5086 | -2,723758 |
| mll8487 | -2,724428 |
| mll0640 | -2,724732 |
| mlr0192 | -2,725046 |
| mlr5406 | -2,725736 |
| mlr3177 | -2,727276 |
| mll1596 | -2,730213 |
| msl7423 | -2,730258 |
| mlr3523 | -2,731904 |
| mll0891 | -2,7332565 |
| mlr7599 | -2,733694 |
| mlr0658 | -2,733865 |
| msr4233 | -2,734398 |
| mll5053 | -2,735628 |
| mll2219 | -2,737799 |
| mlr2930 | -2,739358 |
| mll0798 | -2,744207 |
| mlr6579 | -2,744886 |
| mlr8351 | -2,746845 |
| mll5328 | -2,747891 |
| mlr7143 | -2,748001 |
| mlr3053 | -2,748662 |
| mlr5624 | -2,749946 |
| mlr4366 | -2,755032 |
| msr0259 | -2,758417 |
| mlr8426 | -2,758535 |
| mlr4231 | -2,760199 |
| msr0848 | -2,7608205 |
| mll3520 | -2,761571 |
| mlr5544 | -2,76161 |
| mll1089 | -2,762451 |
| mll1100 | -2,768727 |
| mll2848 | -2,770348 |
| mll1560 | -2,772197 |
| mlr5807 | -2,773596 |
| mlr9240 | -2,773683 |
| mll1103 | -2,773732 |
| mll1628 | -2,77601 |
| mlr7511 | -2,779448 |
| msl8449 | -2,780181 |
| mlr3052 | -2,781402 |
| mlr4354 | -2,783089 |
| mll4000 | -2,783727 |
| mlr8165 | -2,786105 |
| mlr1025 | -2,786807 |
| mll1091 | -2,788282 |
| mlr6950 | -2,788927 |
| mlr2937 | -2,790462 |
| mlr9255 | -2,790754 |
| mlr4570 | -2,790851 |
| mll5492 | -2,796387 |
| mll8234 | -2,796576 |
| mll0631 | -2,797169 |
| mll8309 | -2,797826 |
| mlr8316 | -2,798904 |
| mlr8268 | -2,800648 |
| mll0606 | -2,801926 |
| mlr0386 | -2,805126 |
| mlr7877 | -2,806354 |
| mll7336 | -2,811774 |
| mll0675 | -2,812172 |
| mll0352 | -2,813667 |
| mlr1245 | -2,8218145 |
| mll4260 | -2,824296 |
| mll1108 | -2,827404 |
| mlr6390 | -2,827846 |
| mll1570 | -2,833537 |
| mlr4844 | -2,836088 |
| mll2705 | -2,838262 |
| mlr6536 | -2,847797 |
| mlr0353 | -2,850587 |
| mll1192 | -2,854277 |
| mlr7554 | -2,857373 |
| mll5578 | -2,861535 |
| mll6119 | -2,865656 |
| mll3945 | -2,867287 |
| mlr7555 | -2,868442 |
| mlr4692 | -2,868844 |
| mll0404 | -2,869762 |
| mll8359 | -2,869823 |
| mlr3158 | -2,871311 |
| mll6778 | -2,872072 |
| mlr0321 | -2,873103 |
| mll0911 | -2,874658 |
| mlr8553 | -2,8752555 |
| mll0427 | -2,876675 |
| mlr7687 | -2,879241 |
| mlr3366 | -2,8819195 |
| mll4921 | -2,882568 |
| mlr9250 | -2,88516 |
| mlr0688 | -2,887066 |
| mlr0112 | -2,8886105 |
| mll3558 | -2,889457 |
| mll4267 | -2,891454 |
| mlr7753 | -2,893034 |
| mlr6275 | -2,89311 |
| mlr9239 | -2,893241 |
| mll2248 | -2,89581 |
| mll0140 | -2,897007 |
| mlr6097 | -2,897852 |
| mll2905 | -2,898901 |
| mll2737 | -2,898908 |
| mlr1461 | -2,899617 |
| mlr4826 | -2,901771 |
| msl4331 | -2,901958 |
| mll4405 | -2,902129 |
| mll7863 | -2,904174 |
| mll3194 | -2,908126 |
| mll4592 | -2,908671 |
| mlr5600 | -2,9087825 |
| mll2816 | -2,910373 |
| msr7851 | -2,9108625 |
| mlr5183 | -2,913948 |
| mll3974 | -2,915588 |
| mll4120 | -2,918193 |
| mll5170 | -2,921437 |
| mlr0402 | -2,9240005 |
| mlr5545 | -2,925411 |
| mll0914 | -2,929324 |
| mll4780 | -2,930854 |
| mll0813 | -2,93122 |
| mll7507 | -2,934549 |
| mll6424 | -2,935626 |
| mll3436 | -2,9414175 |
| mll7579 | -2,947847 |
| mll4769 | -2,949211 |
| mlr7410 | -2,949563 |
| mll1354 | -2,950656 |
| mlr7098 | -2,954351 |
| mlr5417 | -2,9561605 |
| msl2078 | -2,957082 |
| mlr8491 | -2,957111 |
| mlr7590 | -2,959056 |
| mll1632 | -2,9613595 |
| mlr7400 | -2,962439 |
| mlr9237 | -2,964668 |
| mlr7548 | -2,966473 |
| mlr8400 | -2,974482 |
| msl4696 | -2,97466 |
| mll0351 | -2,9771385 |
| mll3280 | -2,979422 |
| msr2657 | -2,979506 |
| msr6259 | -2,979563 |
| mlr3474 | -2,979604 |
| mlr3188 | -2,987023 |
| mlr2759 | -2,990583 |
| mll2845 | -2,999203 |
| mlr8425 | -2,999669 |
| mlr7341 | -3,000509 |
| mll2844 | -3,000695 |
| mlr0496 | -3,001072 |
| mll1359 | -3,001289 |
| mlr5487 | -3,002079 |
| mlr0332 | -3,003242 |
| mll7701 | -3,003248 |
| mll0638 | -3,00381 |
| mlr5535 | -3,004362 |
| mlr5073 | -3,004571 |
| mlr3203 | -3,006725 |
| mll1352 | -3,007262 |
| mll5622 | -3,01304 |
| mll8437 | -3,016449 |
| mlr4495 | -3,01809 |
| mll0513 | -3,019154 |
| mll4103 | -3,019216 |
| mlr2915 | -3,019768 |
| mlr3249 | -3,024041 |
| mlr3577 | -3,024461 |
| mll7792 | -3,029488 |
| mlr0198 | -3,029811 |
| mll0185 | -3,031173 |
| msr5525 | -3,0312525 |
| mlr8251 | -3,032309 |
| mlr5029 | -3,033434 |
| mlr7495 | -3,037418 |
| mll2084 | -3,039666 |
| mlr5416 | -3,040193 |
| mlr8255 | -3,041485 |
| mll1555 | -3,04326 |
| mll2527 | -3,046493 |
| msr7654 | -3,047864 |
| mlr5594 | -3,048358 |
| mll1557 | -3,0487605 |
| mlr3041 | -3,048915 |
| mll6452 | -3,04905 |
| mll0605 | -3,049801 |
| mlr8310 | -3,051911 |
| mlr5562 | -3,05273 |
| mll7836 | -3,05314 |
| mll3975 | -3,054457 |
| mlr8358 | -3,056166 |
| mlr4627 | -3,056918 |
| mlr2685 | -3,0576 |
| mlr7498 | -3,059353 |
| mll5126 | -3,064185 |
| mll1358 | -3,06508 |
| mlr7550 | -3,06886 |
| mlr3248 | -3,069064 |
| mlr0045 | -3,075281 |
| msl7460 | -3,075326 |
| mlr1064 | -3,0764125 |
| mlr5264 | -3,082393 |
| mlr7432 | -3,084266 |
| mlr0334 | -3,08486 |
| mll8261 | -3,085059 |
| mll0656 | -3,085225 |
| mll8554 | -3,086034 |
| mlr5598 | -3,090998 |
| mll2811 | -3,091032 |
| mll2286 | -3,096116 |
| msl0044 | -3,096416 |
| mlr2896 | -3,0980495 |
| msl5090 | -3,099615 |
| mll6895 | -3,102328 |
| mlr2913 | -3,105312 |
| mll1549 | -3,106402 |
| mll4308 | -3,110376 |
| mlr3486 | -3,11142 |
| mll6628 | -3,112899 |
| mlr8490 | -3,113056 |
| mlr7552 | -3,115699 |
| mlr8188 | -3,118219 |
| mll6586 | -3,12282 |
| msr8736 | -3,124646 |
| mll5344 | -3,125107 |
| mlr4660 | -3,128196 |
| msl0755 | -3,132421 |
| mll7791 | -3,13641 |
| mlr3921 | -3,137108 |
| mll5345 | -3,137188 |
| mll0727 | -3,142464 |
| mlr7411 | -3,143761 |
| mll0056 | -3,152601 |
| mlr7763 | -3,154142 |
| mll3888 | -3,155757 |
| mll7866 | -3,1557645 |
| mlr4737 | -3,157736 |
| msr1398 | -3,1580125 |
| mll1467 | -3,158355 |
| mll2707 | -3,1628205 |
| mll0079 | -3,167949 |
| mll3161 | -3,170972 |
| mlr7894 | -3,171793 |
| mlr8423 | -3,172318 |
| mll7508 | -3,173979 |
| mlr4641 | -3,17649 |
| mll1624 | -3,178394 |
| mlr3958 | -3,181714 |
| mlr0220 | -3,183499 |
| mlr8433 | -3,185547 |
| mlr4779 | -3,187382 |
| mlr7593 | -3,189901 |
| mlr7257 | -3,1957745 |
| mll0426 | -3,196713 |
| mlr4635 | -3,201788 |
| mll1468 | -3,203214 |
| mll5580 | -3,206623 |
| mlr6576 | -3,207306 |
| mll9373 | -3,210396 |
| mll6598 | -3,21072 |
| mll3972 | -3,2109205 |
| mlr2709 | -3,212204 |
| mll1551 | -3,215897 |
| mlr7435 | -3,215978 |
| mlr7850 | -3,225084 |
| mlr2931 | -3,226453 |
| mlr2695 | -3,226475 |
| mll3887 | -3,232586 |
| mlr7441 | -3,232765 |
| mll5582 | -3,232819 |
| mll0635 | -3,235857 |
| mlr9259 | -3,237082 |
| mlr4777 | -3,237626 |
| mlr8151 | -3,246138 |
| mlr8156 | -3,25214 |
| mll1390 | -3,252444 |
| mll1078 | -3,253063 |
| mll1559 | -3,260681 |
| mll1446 | -3,2653 |
| mll3819 | -3,265349 |
| mlr2806 | -3,265813 |
| mll2891 | -3,272235 |
| mlr6537 | -3,277304 |
| mlr3490 | -3,277333 |
| mlr2923 | -3,282276 |
| mlr3016 | -3,282616 |
| mlr0528 | -3,283353 |
| mll8546 | -3,285574 |
| msr1490 | -3,290749 |
| msl6453 | -3,291352 |
| mlr7434 | -3,293549 |
| mlr1674 | -3,296299 |
| msl3782 | -3,297404 |
| mll4767 | -3,299919 |
| mll2887 | -3,3030645 |
| msl3456 | -3,305064 |
| mll5060 | -3,306205 |
| mlr5215 | -3,307866 |
| mll1565 | -3,308821 |
| mll4075 | -3,309179 |
| mlr7491 | -3,309747 |
| mlr5146 | -3,31049 |
| mll1309 | -3,311015 |
| mll4765 | -3,312628 |
| mll3593 | -3,3128555 |
| msr0050 | -3,315349 |
| mlr3120 | -3,3157835 |
| mll1366 | -3,316204 |
| mll5672 | -3,31659 |
| mlr0550 | -3,320956 |
| mll8296 | -3,321485 |
| mlr5508 | -3,325797 |
| mlr2912 | -3,328123 |
| mlr6631 | -3,33184 |
| mll0525 | -3,33349 |
| mlr7496 | -3,334526 |
| mlr7709 | -3,33628 |
| mll5055 | -3,343093 |
| mlr7497 | -3,343219 |
| mlr4711 | -3,3432315 |
| mlr7598 | -3,346108 |
| mll4552 | -3,34745 |
| mlr0666 | -3,349838 |
| mll4541 | -3,358318 |
| mll0203 | -3,358816 |
| mll7834 | -3,36231 |
| mll4122 | -3,364802 |
| mll5251 | -3,367127 |
| mll1355 | -3,367283 |
| mlr5595 | -3,3741095 |
| mll3713 | -3,374363 |
| mll4927 | -3,375711 |
| mlr2541 | -3,37745 |
| mll5027 | -3,377666 |
| mll3977 | -3,380205 |
| mll0725 | -3,381867 |
| mll5591 | -3,382629 |
| mll2878 | -3,383465 |
| mlr1935 | -3,387347 |
| mlr2925 | -3,389134 |
| mll1543 | -3,391157 |
| mlr2815 | -3,392898 |
| mll7516 | -3,405257 |
| mll0207 | -3,407694 |
| mll1371 | -3,413265 |
| mlr1663 | -3,416858 |
| mlr7493 | -3,417852 |
| mll5346 | -3,419506 |
| mlr8394 | -3,41959 |
| mll1207 | -3,423334 |
| mlr0372 | -3,423379 |
| mlr4622 | -3,423502 |
| mlr0809 | -3,42749 |
| mll8145 | -3,428155 |
| mll0634 | -3,429191 |
| mll6624 | -3,431429 |
| mlr6847 | -3,4316635 |
| mlr7927 | -3,435993 |
| mll4073 | -3,436809 |
| mlr6682 | -3,438779 |
| mll0202 | -3,441678 |
| mll4925 | -3,443568 |
| mll0642 | -3,445902 |
| mlr7746 | -3,449758 |
| mll5113 | -3,4640575 |
| msr4160 | -3,473459 |
| mlr1176 | -3,473671 |
| mlr7525 | -3,477997 |
| mlr0168 | -3,492436 |
| mll0361 | -3,493991 |
| mlr7558 | -3,496707 |
| mll0533 | -3,502292 |
| mll3477 | -3,503364 |
| mlr3476 | -3,507569 |
| mlr2613 | -3,5101505 |
| mll5024 | -3,518469 |
| mll3110 | -3,523247 |
| mlr7156 | -3,524523 |
| mll2890 | -3,53113 |
| mll3886 | -3,534247 |
| mll0643 | -3,534837 |
| mlr3165 | -3,536006 |
| mll0936 | -3,545353 |
| msl2821 | -3,546744 |
| mlr4624 | -3,5494565 |
| mlr4089 | -3,552924 |
| mll6639 | -3,554241 |
| mlr0328 | -3,554432 |
| mll1107 | -3,557355 |
| mlr2927 | -3,565817 |
| mlr5176 | -3,568865 |
| msl3109 | -3,572676 |
| mll4766 | -3,58433 |
| mll1646 | -3,584609 |
| msr2943 | -3,590496 |
| msl3438 | -3,594738 |
| mlr4640 | -3,60351 |
| mll1367 | -3,606618 |
| mll5669 | -3,613658 |
| mll8419 | -3,620189 |
| mll6524 | -3,621426 |
| mll7962 | -3,62683 |
| mll1558 | -3,632384 |
| mll1768 | -3,6457 |
| mll1054 | -3,647725 |
| mlr4953 | -3,647726 |
| mll6629 | -3,650157 |
| mll5025 | -3,652365 |
| mll1072 | -3,653137 |
| mlr2917 | -3,656618 |
| mll3296 | -3,670772 |
| mlr7490 | -3,671221 |
| mlr2964 | -3,675609 |
| mll1357 | -3,677269 |
| mlr7650 | -3,683926 |
| mlr2928 | -3,688159 |
| mlr7408 | -3,694599 |
| mll1546 | -3,699101 |
| mlr6849 | -3,699713 |
| mll0680 | -3,70538 |
| mll6844 | -3,707029 |
| mll1308 | -3,711166 |
| mll3968 | -3,716353 |
| mlr6581 | -3,718048 |
| msl6623 | -3,750227 |
| mll4066 | -3,750517 |
| mll8436 | -3,751725 |
| mll7565 | -3,752034 |
| mll4303 | -3,7538645 |
| msr5347 | -3,759564 |
| mll1372 | -3,761896 |
| mlr6848 | -3,764431 |
| mlr3185 | -3,765376 |
| mlr4774 | -3,768158 |
| mll5581 | -3,76871 |
| mlr6471 | -3,769254 |
| mlr6533 | -3,833633 |
| mlr2940 | -3,845394 |
| mlr0215 | -3,848012 |
| mlr0754 | -3,852171 |
| mlr5418 | -3,868483 |
| mll2706 | -3,87281 |
| mll5916 | -3,915878 |
| mll7917 | -3,919289 |
| mll3967 | -3,932223 |
| msr3814 | -3,9401515 |
| mll4304 | -3,953766 |
| mll1563 | -3,981754 |
| mlr8418 | -3,9916285 |
| mlr6585 | -4,006251 |
| mlr4158 | -4,011979 |
| mll6630 | -4,036091 |
| mlr6534 | -4,0502495 |
| mll1550 | -4,054792 |
| msr6604 | -4,054833 |
| mll1077 | -4,056186 |
| mll0212 | -4,060068 |
| mlr1655 | -4,078942 |
| msr7494 | -4,096005 |
| mll5410 | -4,1062785 |
| mlr4772 | -4,130802 |
| mlr5313 | -4,172752 |
| mll5695 | -4,199518 |
| mll6600 | -4,235049 |
| mlr4720 | -4,273818 |
| mlr2911 | -4,305835 |
| mlr5349 | -4,392104 |
| mll3878 | -4,403956 |
| mll0636 | -4,419633 |
| mlr7520 | -4,4322265 |
| mll6587 | -4,48991 |
| mll1752 | -4,717385 |
| mll6577 | -5,07187 |
| mll6578 | -5,107432 |
